# Supplementary material for: Bacterial genome size and gene functional diversity negatively correlate with taxonomic diversity along a pH gradient
Source: Nat Commun. 2023 Nov 17;14:7437. doi: 10.1038/s41467-023-43297-w (PMC10656551; doi:10.1038/s41467-023-43297-w)
Supplement: Supplementary file 1 — Supplementary Information [file 41467_2023_43297_MOESM1_ESM.pdf]

Supplementary Information for “Bacterial genome size and gene functional diversity negatively correlate with taxonomic diversity along a pH gradient” by Wang et al.

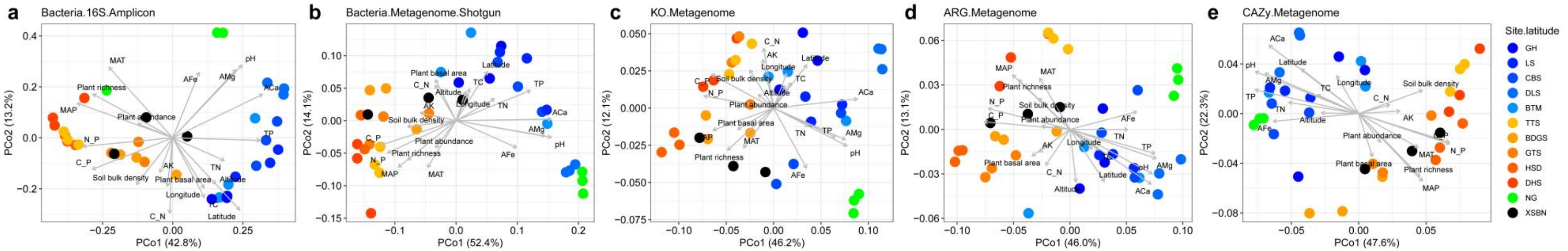

a

	Dim1	Dim2	r2	Pr(>r)	
Latitude	0.64434	−0.76474	0.7235	0.001	***
Longitude	0.49235	−0.87040	0.2699	0.004	**
Altitude	0.64390	−0.76511	0.2947	0.006	**
MAP	−0.92840	0.37159	0.7042	0.001	***
MAT	−0.63470	0.77276	0.6747	0.001	***
TC	0.43316	−0.90132	0.3550	0.003	**
TN	0.89899	−0.43797	0.2195	0.027	*
TP	0.99948	−0.03216	0.5093	0.001	***
pH	0.65402	0.75648	0.7467	0.001	***
ACa	0.88251	0.47029	0.6914	0.001	***
AMg	0.64494	0.76423	0.6420	0.001	***
AFe	0.34361	0.93911	0.3927	0.001	***
AK	−0.32032	−0.94731	0.1072	0.150	
C_N	−0.03667	−0.99933	0.4605	0.001	***
C_P	−0.91840	−0.39565	0.5110	0.001	***
N_P	−0.99449	−0.10488	0.5432	0.001	***
Soil bulk density	−0.57728	−0.81655	0.2642	0.006	**
Plant abundance	−0.91311	0.40772	0.0973	0.181	
Plant basal area	−0.09837	−0.99515	0.1916	0.035	*
Plant richness	−0.85613	0.51676	0.5796	0.001	***

b

	Dim1	Dim2	r2	Pr(>r)	
Latitude	0.42080	0.90715	0.5000	0.001	***
Longitude	0.31234	0.94997	0.1370	0.079	.
Altitude	0.23115	0.97292	0.1535	0.067	.
MAP	−0.67158	−0.74093	0.6670	0.001	***
MAT	−0.40495	−0.91434	0.4457	0.002	**
TC	0.30022	0.95387	0.1873	0.029	*
TN	0.78687	0.61712	0.2139	0.024	*
TP	0.87349	0.48684	0.6527	0.001	***
pH	0.97217	−0.23426	0.8676	0.001	***
ACa	0.99977	0.02166	0.8189	0.001	***
AMg	0.97346	−0.22886	0.7548	0.001	***
AFe	0.85316	−0.52164	0.4543	0.001	***
AK	−0.95701	0.29007	0.1260	0.116	
C_N	−0.39574	0.91836	0.2544	0.008	**
C_P	−0.91325	−0.40740	0.5970	0.001	***
N_P	−0.83879	−0.54446	0.6023	0.001	***
Soil bulk density	−0.98603	−0.16654	0.2178	0.021	*
Plant abundance	−0.48348	−0.87535	0.1636	0.048	*
Plant basal area	−0.34464	0.93873	0.3233	0.004	**
Plant richness	−0.66758	−0.74453	0.4741	0.001	***

c

	Dim1	Dim2	r2	Pr(>r)	
Latitude	0.58576	0.81049	0.3834	0.001	***
Longitude	0.33285	0.94298	0.1808	0.038	*
Altitude	0.44348	0.89628	0.0827	0.222	
MAP	−0.93189	−0.36274	0.5043	0.001	***
MAT	−0.57871	−0.81554	0.3213	0.004	**
TC	0.54793	0.83652	0.1222	0.104	
TN	0.87435	−0.48529	0.1904	0.028	*
TP	0.89319	−0.44967	0.5693	0.001	***
pH	0.87604	−0.48223	0.8325	0.001	***
ACa	0.99333	0.11529	0.8480	0.001	***
AMg	0.90581	−0.42369	0.7441	0.001	***
AFe	0.45755	−0.88919	0.6142	0.001	***
AK	−0.20527	0.97871	0.3309	0.003	**
C_N	−0.15276	0.98826	0.4927	0.001	***
C_P	−0.86484	0.50206	0.6099	0.001	***
N_P	−0.98352	0.18080	0.5977	0.001	***
Soil bulk density	−0.50795	0.86139	0.2628	0.007	**
Plant abundance	−0.72252	0.69135	0.0527	0.399	
Plant basal area	−0.84294	−0.53801	0.2144	0.020	*
Plant richness	−0.71641	−0.69768	0.4920	0.001	***

d

	Dim1	Dim2	r2	Pr(>r)	
Latitude	0.48603	−0.87394	0.4106	0.001	***
Longitude	0.69607	−0.71798	0.0854	0.219	
Altitude	0.09595	−0.99539	0.3231	0.005	**
MAP	−0.66337	0.74829	0.6141	0.001	***
MAT	−0.44612	0.89497	0.3772	0.002	**
TC	0.76116	−0.64856	0.1173	0.112	
TN	0.99999	−0.00390	0.2730	0.007	**
TP	0.94737	−0.32014	0.6591	0.001	***
pH	0.84127	−0.54061	0.7804	0.001	***
ACa	0.78041	−0.62527	0.8867	0.001	***
AMg	0.92829	−0.37186	0.7052	0.001	***
AFe	0.95377	0.30055	0.4009	0.001	***
AK	−0.75505	−0.65567	0.1376	0.085	.
C_N	−0.49333	−0.86984	0.1969	0.032	*
C_P	−0.99442	0.10552	0.6067	0.001	***
N_P	−0.95648	0.29179	0.5950	0.001	***
Soil bulk density	−0.64732	0.76222	0.1931	0.023	*
Plant abundance	−0.99126	0.13192	0.0668	0.329	
Plant basal area	−0.76782	−0.64067	0.1976	0.031	*
Plant richness	−0.65745	0.75350	0.5007	0.001	***

e

	Dim1	Dim2	r2	Pr(>r)	
Latitude	−0.72585	0.68786	0.3686	0.002	**
Longitude	−0.43210	0.90182	0.1540	0.055	.
Altitude	−0.99984	0.01814	0.0825	0.250	
MAP	0.61327	−0.78987	0.6373	0.001	***
MAT	0.82086	−0.57114	0.3090	0.008	**
TC	−0.87596	0.48238	0.1324	0.084	.
TN	−0.98160	0.19094	0.2999	0.005	**
TP	−0.96307	0.26925	0.6831	0.001	***
pH	−0.89688	0.44227	0.7826	0.001	***
ACa	−0.73360	0.67958	0.8802	0.001	***
AMg	−0.88603	0.46363	0.6596	0.001	***
AFe	−0.99401	−0.10928	0.4367	0.001	***
AK	0.99703	0.07696	0.1005	0.181	
C_N	0.94472	0.32788	0.0635	0.344	
C_P	0.93341	−0.35880	0.5566	0.001	***
N_P	0.92667	−0.37588	0.5655	0.001	***
Soil bulk density	0.91417	0.40533	0.2858	0.005	**
Plant abundance	0.53150	−0.84706	0.1000	0.176	
Plant basal area	0.25323	−0.96741	0.1722	0.052	.
Plant richness	0.62642	−0.77948	0.5625	0.001	***

Supplementary Fig. 1. Bacterial community and functional composition in association with environmental variables. a-b Principal coordinate (PCo) analysis with environmental fitting (envfit) showing significant association of **a** amplicon-based and **b** metagenome-based bacterial community composition with soil pH as well as other biotic and abiotic variables. Note **a** and **b** are the same as Fig. 1b and Fig. 1c with additional results of envfit. **c-e** PCo analysis and envfit showing significant association of soil pH as well as other biotic and abiotic variables with several groups of functional genes: **c** Kyoto Encyclopedia of Genes and Genomes Ontology (KO), **d** antibiotic resistance genes (ARG), and **e** carbohydrate-active enzymes (CAZy). MAP: mean annual precipitation; MAT: mean annual temperature; TC: total carbon; TN: total nitrogen; TP: total phosphorus; ACa: available calcium; AMg: available magnesium; AFe: available iron; AK: available potassium; C_N: carbon nitrogen

ratio; C_P: carbon phosphorus ratio; N_P: nitrogen phosphorus ratio. n = 36 samples. The numerical data is available in Supplementary Data 1. Source data are provided as a Source Data file.

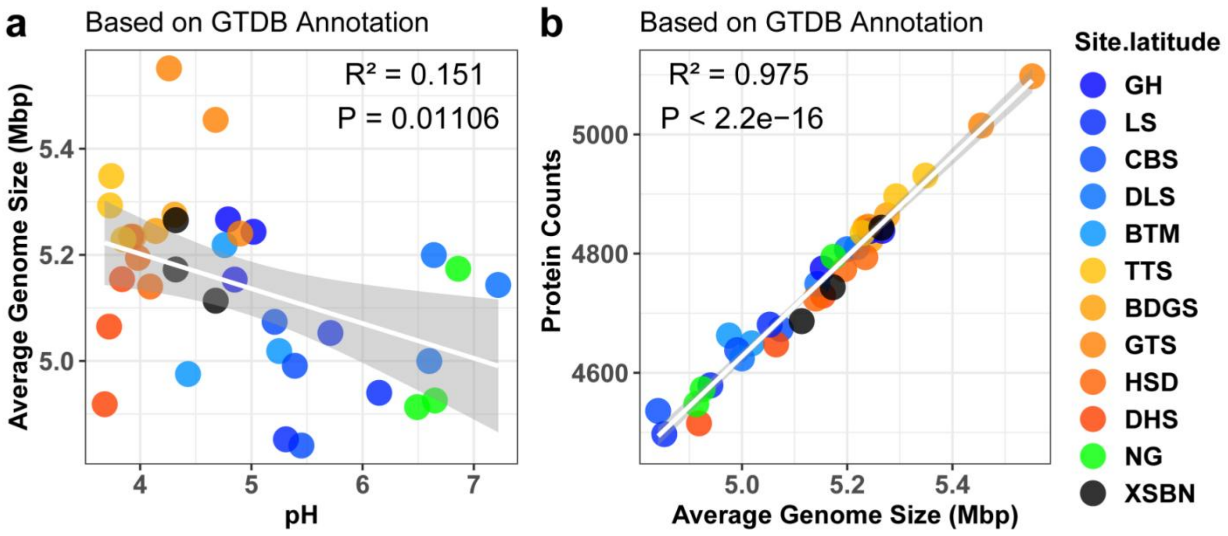

Supplementary Fig. 2. Genomic traits along a pH gradient. Bacterial average genome size and protein counts per genome are calculated by annotating 16S rRNA gene sequence to Genome Taxonomy Database (GTDB). **a** Bacterial average genome size decreased as pH changed from acidic to neutral. **b** Bacterial protein counts per genome significantly and positively correlate with average genome size. Linear regression model with two-sided test was used for the statistical analysis, and adjusted R-squared was used. n = 36 samples. The grey area around the smooth line indicates the 95% confidence interval. Source data are provided as a Source Data file.

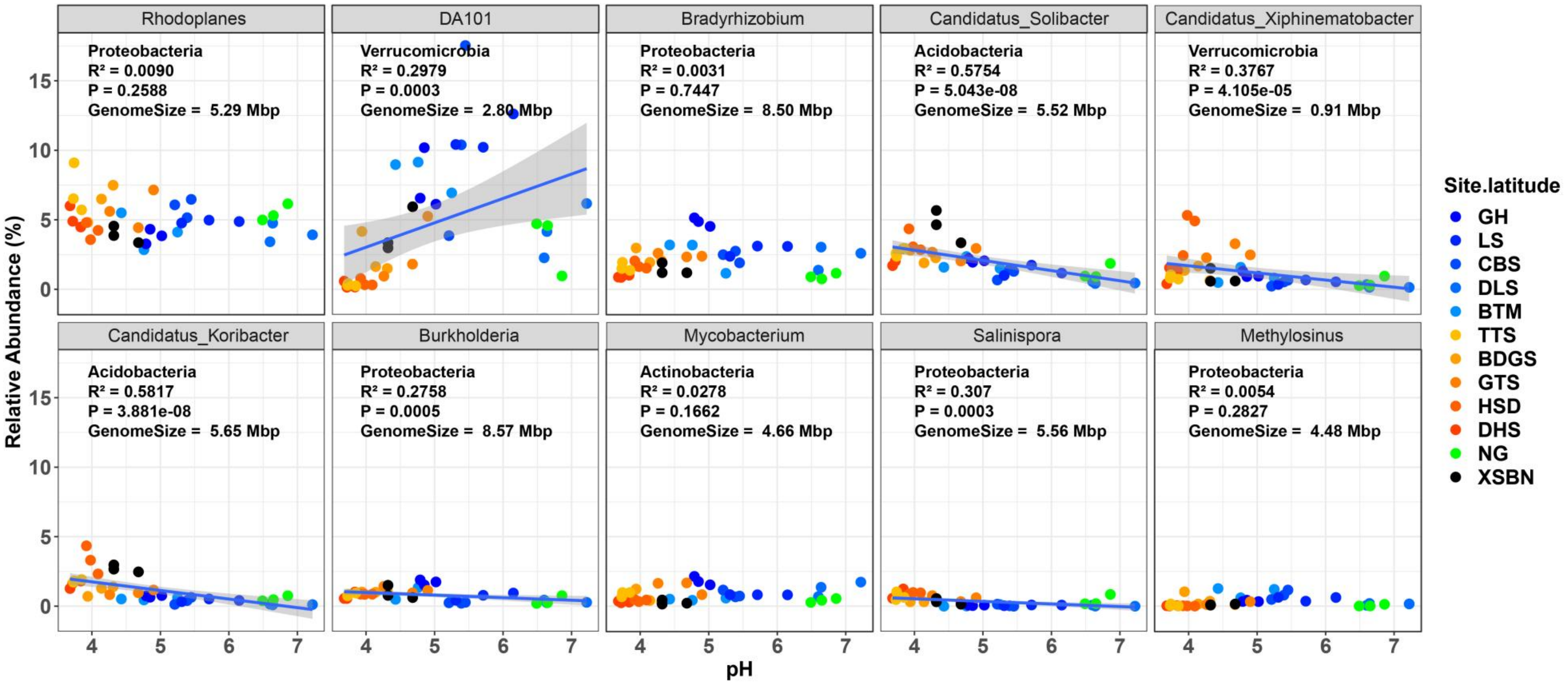

Supplementary Fig. 3. Associations of relative abundance of top 10 bacterial genera with soil pH. The average genome size for each genus is searched from GTDB, NCBI or published literature [49]. Linear regression model with two-sided test was used for the statistical analysis, and adjusted R-squared was used. n = 36 samples. The grey area around the smooth line indicates the 95% confidence interval. Source data are provided as a Source Data file.

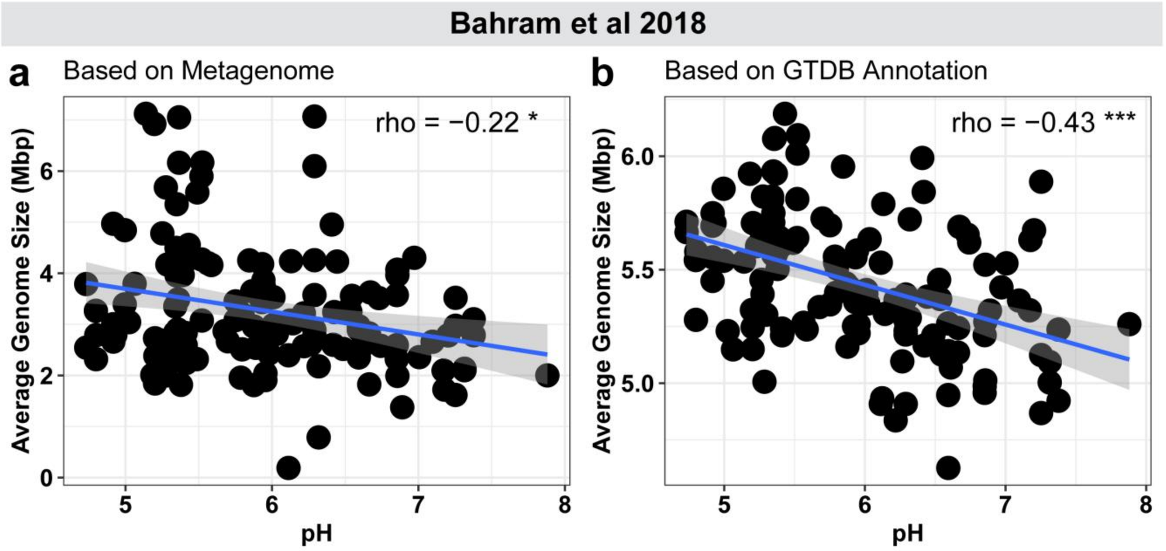

Supplementary Fig. 4. Re-analysis of Bahram et al 2018 showing associations of bacterial average genome size with soil pH. a Soil pH negatively correlated with average genome size as detected by shotgun metagenome using MicrobeCensus pipeline. **b** Soil pH negatively correlated with average genome size as detected by 16S rRNA amplicon referencing Genome Taxonomy Database (GTDB). Linear regression model with two-sided test was used for the statistical analysis, and adjusted R-squared was used. n = 134 samples. The grey area around the smooth line indicates the 95% confidence interval. Source data are provided as a Source Data file.

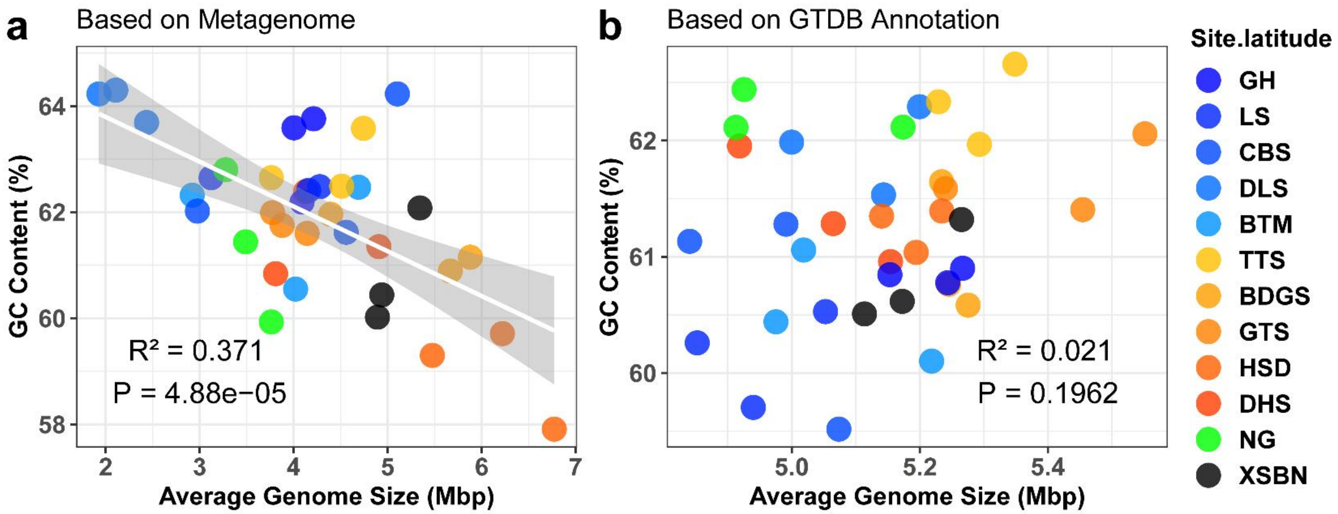

Supplementary Fig. 5. Relationships between bacterial average genome size and GC content (GC%). a-b Metagenome-based analysis **a** showed that bacterial average genome size was negatively correlated with GC%, while the 16S rRNA-GTDB method **b** found that bacterial average genome size was not significantly correlated with GC%. Linear regression model with two-sided test was used for the statistical analysis, and adjusted R-squared was used. n = 36 samples. The grey area around the smooth line indicates the 95% confidence interval. Source data are provided as a Source Data file.

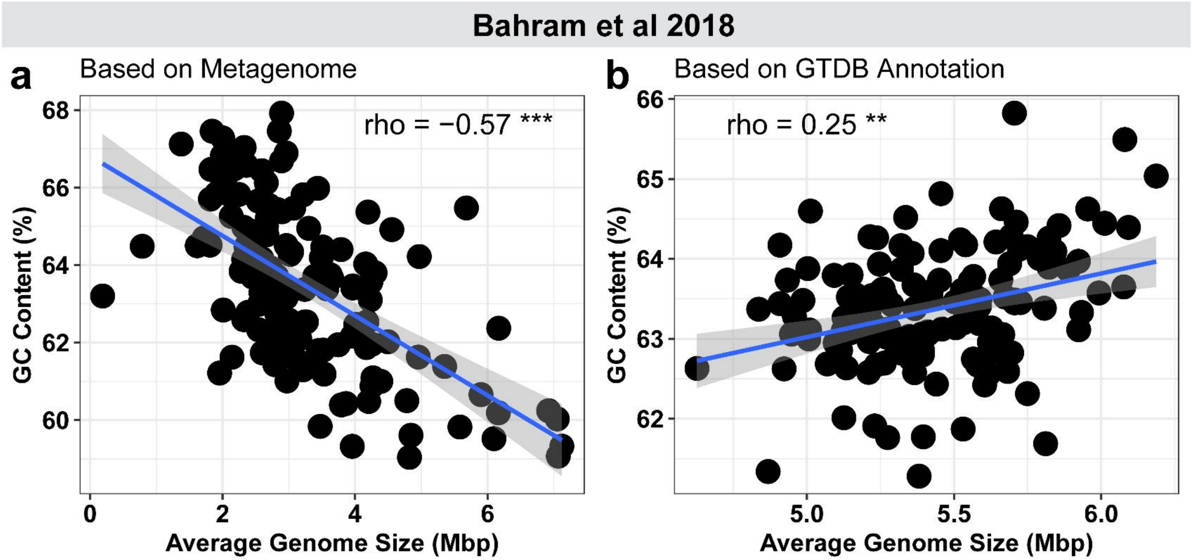

Supplementary Fig. 6. Relationships between bacterial average genome size and GC content (GC%). a-b Re-analysis on the global data of Bahram et al 2018 showed that **a** metagenome-based bacterial average genome size was negatively correlated with GC%, whereas **b** 16S rRNA-GTDB based bacterial average genome size was significantly positively correlated with GC%. Linear regression model with two-sided test was used for the statistical analysis, and adjusted R-squared was used. n = 134 samples. The grey area around the smooth line indicates the 95% confidence interval. Source data are provided as a Source Data file.

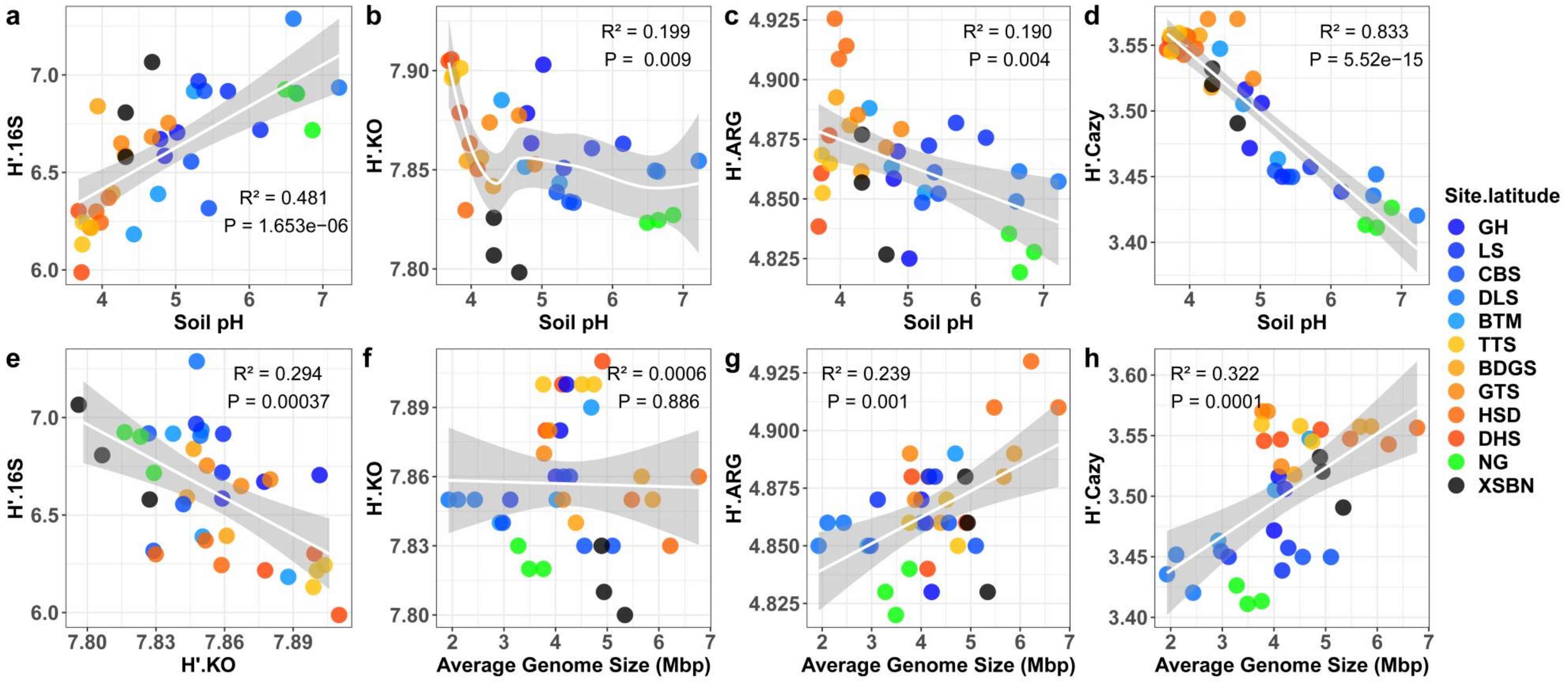

Supplementary Fig. 7. Contrasting distribution patterns for Shannon diversities of bacterial taxonomy and function along pH gradient. Diversities shown are measured by Shannon's index (H'), and diversities measured by richness (S) are provided in Fig. 3. **a Bacterial taxonomic diversity (H'.16S) increased as soil pH changed from acid to neutral.** Bacterial operational taxonomic units (OTUs) are detected by 16S rRNA gene amplicon metabarcoding sequencing. **b Bacterial functional diversity (H'.KO) decreased as pH changed from acid to neutral.** Bacterial functions are determined from the shotgun metagenome, as annotated by Kyoto Encyclopedia of Genes and Genomes (KEGG) Ontology (KO). **c**

Bacterial diversity of antibiotic resistance genes (H'.ARG) decreased as pH changed from acid to neutral. Bacterial antibiotic resistance genes are detected based on shotgun metagenome annotated by the Resfam database. **d Bacterial diversity of carbohydrate-active enzymes (H'.Cazy) genes decreased as pH changed from acid to neutral.** Bacterial carbohydrate-active enzymes genes are detected based on shotgun metagenome annotated by database of CAZy. **e Bacterial taxonomic diversity (H'.16S) negatively correlated with functional gene diversity (H'.KO). f-h** Bacterial average genome size (AGS) positively correlated with functional diversities as measured by **g** H'.ARG and **h** H'.Cazy, and not **f** H'.KO. Linear regression model with two-sided test was used for the statistical analysis, and adjusted R-squared was used. n = 36 samples. The grey area around the smooth line indicates the 95% confidence interval. Source data are provided as a Source Data file.

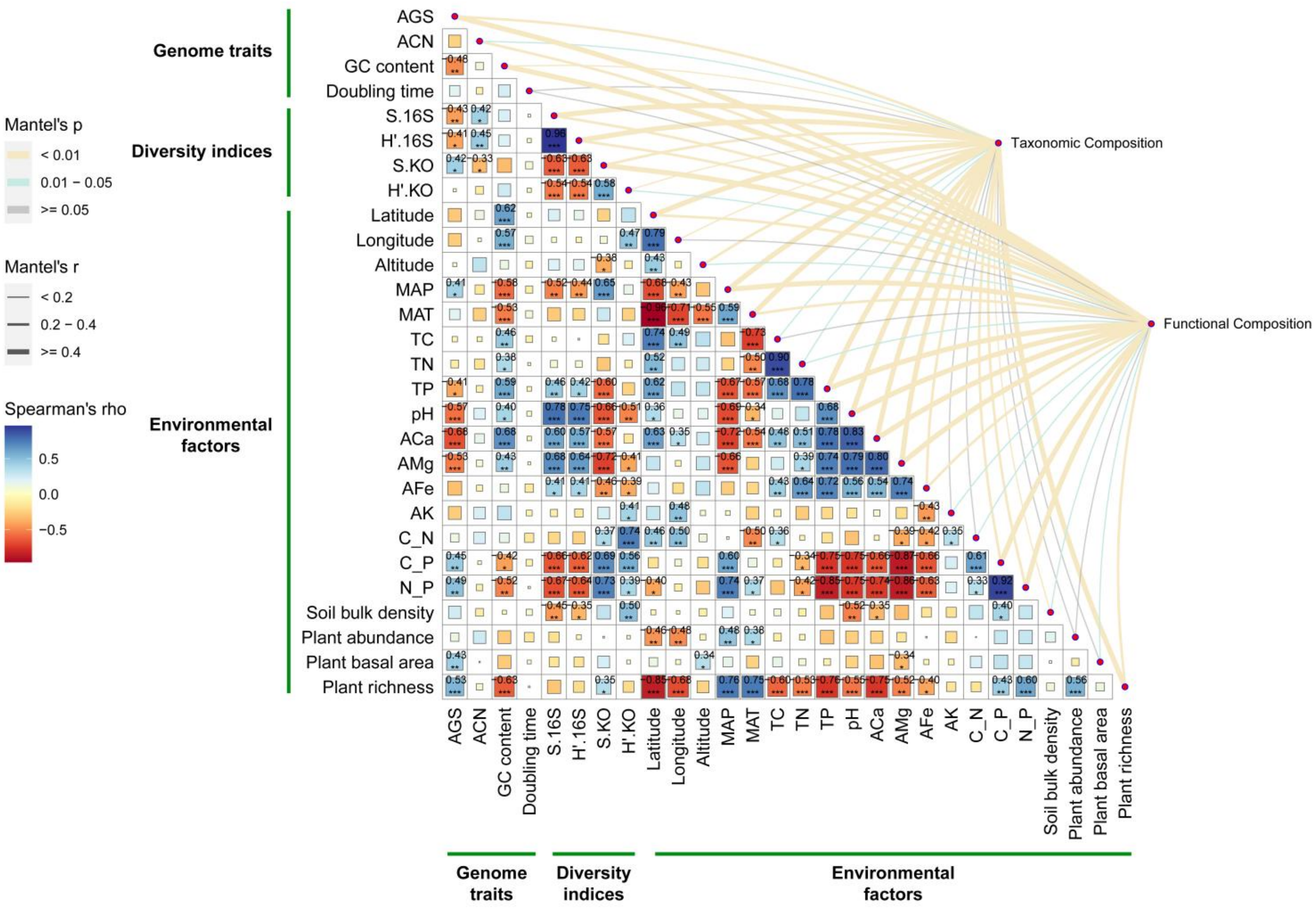

Supplementary Fig. 8. Bacterial taxonomic and functional compositions in association with biotic and abiotic variables. The heatmap in left-bottom triangle showing the intercorrelations among microbial genomic traits, microbial diversity indices, plant, soil and geographical

variables as detected by Spearman's correlation analysis, with a color gradient (red to blue) and box size denoting Spearman's correlation coefficients (rho). The significance of Spearman's correlation is denoted by * $P < 0.05$, ** $P < 0.01$, *** $P < 0.001$. The curved lines in the top-right triangle show the association of bacterial taxonomic (the right-top dot) and functional (the right-bottom dot) compositions with biotic and abiotic variables, as detected by Mantel tests. The width of curved lines corresponds to the r statistic for Mantel test, and the color of curved lines denotes the statistical significance. AGS: average genome size; ACN: average 16S rRNA gene copy number; Doubling time: minimal doubling time; MAP: mean annual precipitation; MAT: mean annual temperature; TC: total carbon; TN: total nitrogen; TP: total phosphorus; ACa: available calcium; AMg: available magnesium; AFe: available iron; AK: available potassium; C_N: carbon nitrogen ratio; C_P: carbon phosphorus ratio; N_P: nitrogen phosphorus ratio. Linear regression model with two-sided test was used for the statistical analysis, and adjusted R-squared was used. n = 36 samples. Source data are provided as a Source Data file.

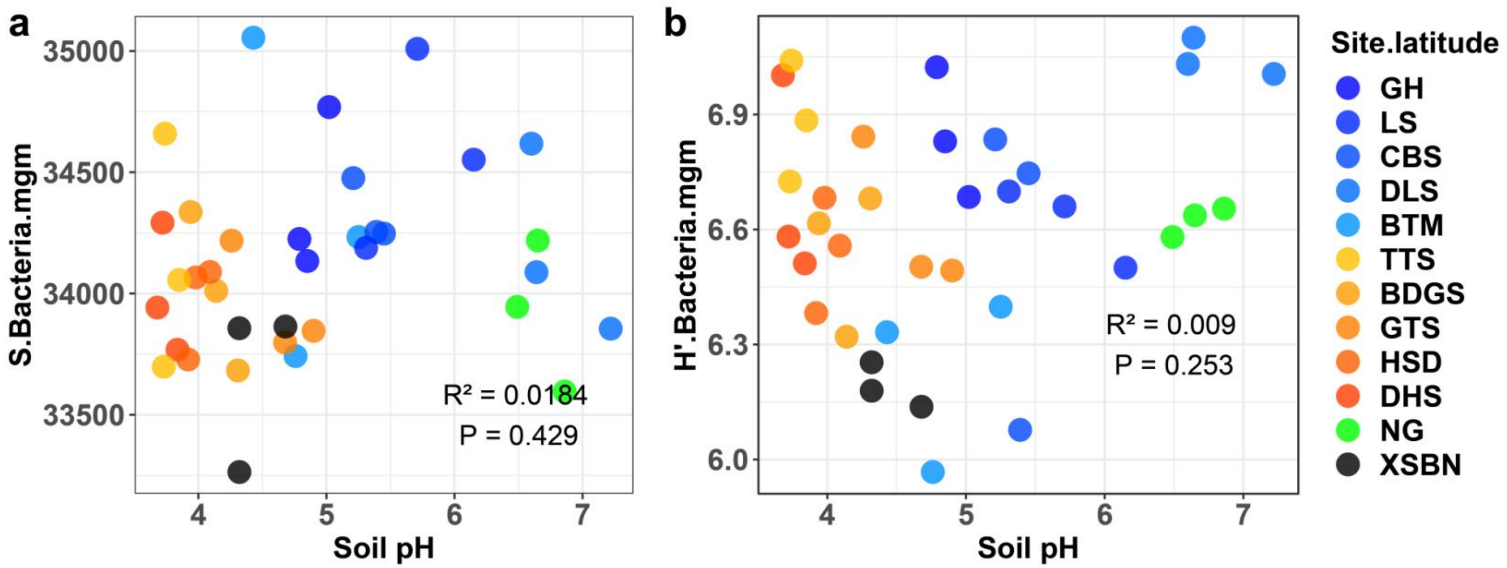

Supplementary Fig. 9. Bacterial taxonomic diversity based on metagenomics data, as measured by **a** richness (S) and **b** Shannon's index (H'), did not change significantly as soil pH changed from acidic to neutral. The bacterial taxonomic diversity was detected from metagenome using Kaiju annotation. Linear regression model with two-sided test was used for the statistical analysis, and adjusted R-squared was used. n = 36 samples. Source data are provided as a Source Data file.

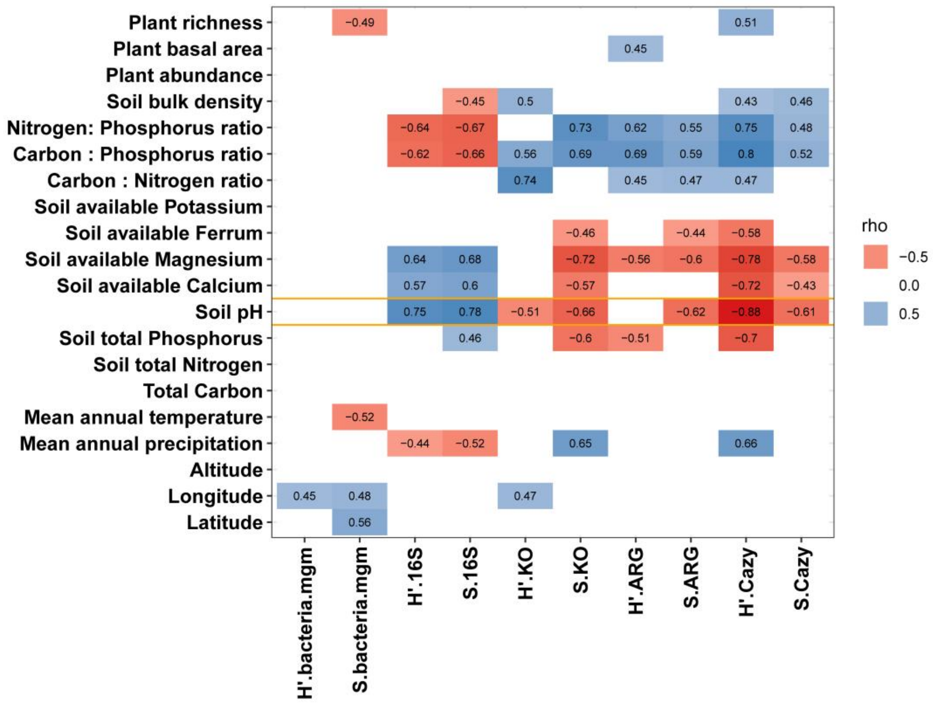

Supplementary Fig. 10. Heatmap showing significant, positive (blue), and negative (red) Spearman's correlations (rho) between environmental factors (left) and indices of bacterial diversities (bottom), as measured by Shannon's index (H') and richness (S). Diversities of bacterial community were both detected by 16S rRNA amplicon (16S) and shotgun metagenome (mgm) annotated by Kaiju method. Diversities of bacterial functions were detected by shotgun metagenome annotated by Kyoto Encyclopedia of Genes and Genomes Ontology (KO), antibiotic resistance genes (ARG), and carbohydrate-active enzymes (CAZy). The values of significant Spearman's correlations (rho) are labeled on the box. Non-significant correlations are colored white. n = 36 samples. Source data are provided as a Source Data file.

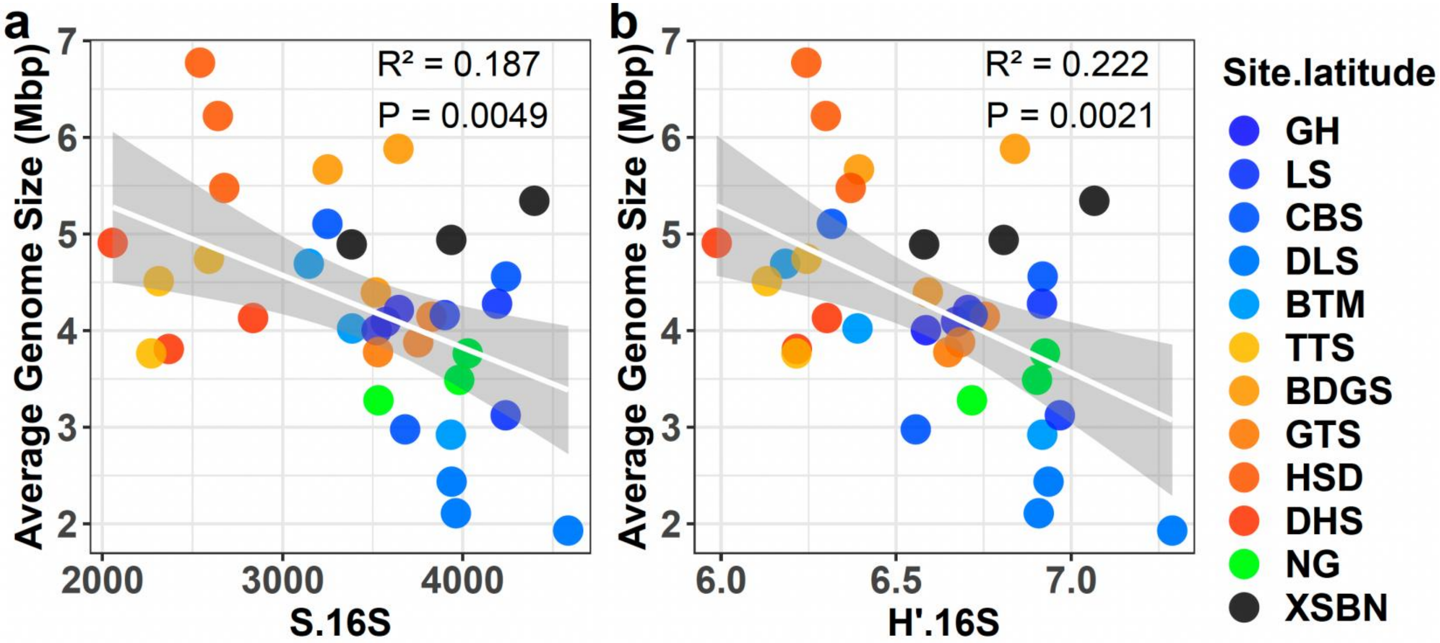

Supplementary Fig. 11. Bacterial average genome size negatively correlated with **a** richness (S.16S) and **b** Shannon diversity (H'.16S) of bacterial community. Linear regression model with two-sided test was used for the statistical analysis, and adjusted R-squared was used. n = 36 samples. The grey area around the smooth line indicates the 95% confidence interval. Source data are provided as a Source Data file.

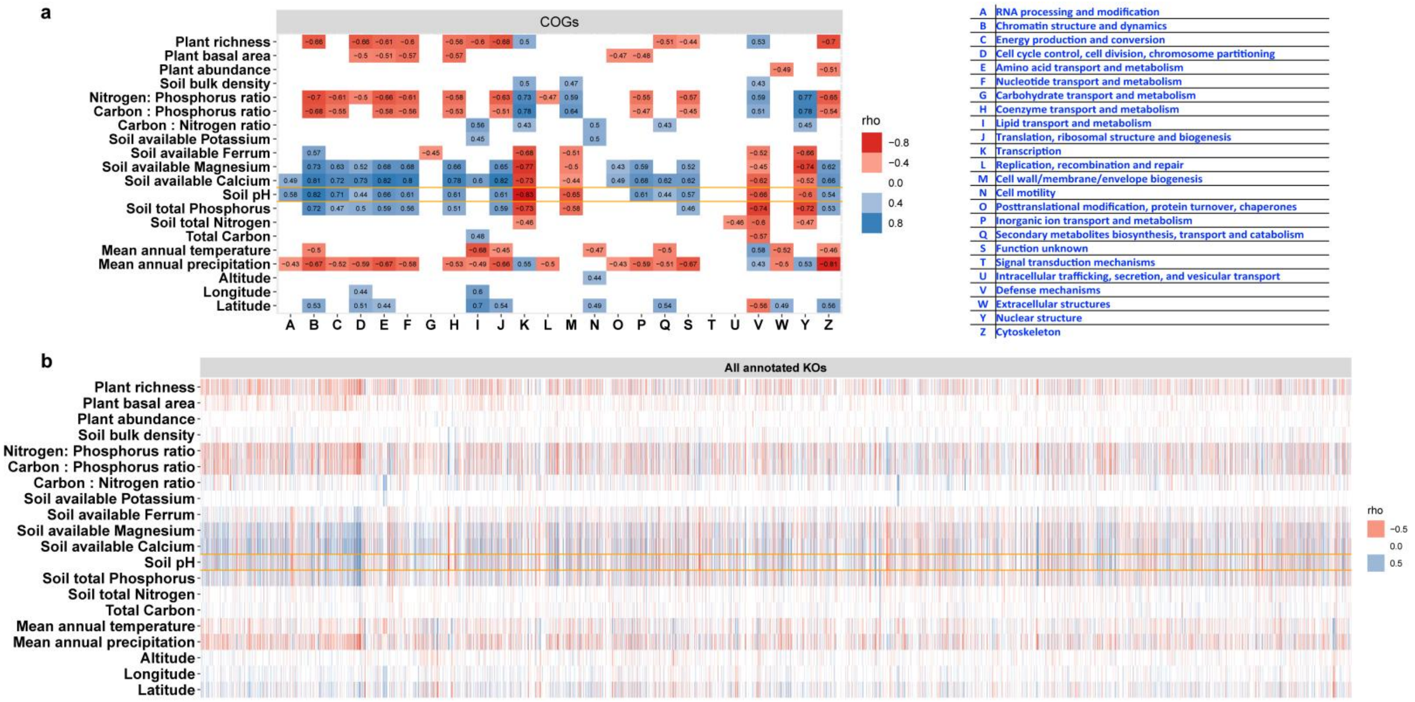

Supplementary Fig. 12. Heatmap showing significant, positive (blue), and negative (red) Spearman's correlations (rho) between environmental variables (left) and metagenome annotated by **a** Clusters of Orthologous Genes (COGs) and **b** Kyoto Encyclopedia of Genes and Genomes Ontology (KO). Annotation information of the 11,065 KOs in the horizontal axis is provided in the source data. Non-significant correlations are colored white. n = 36 samples. Source data are provided as a Source Data file.

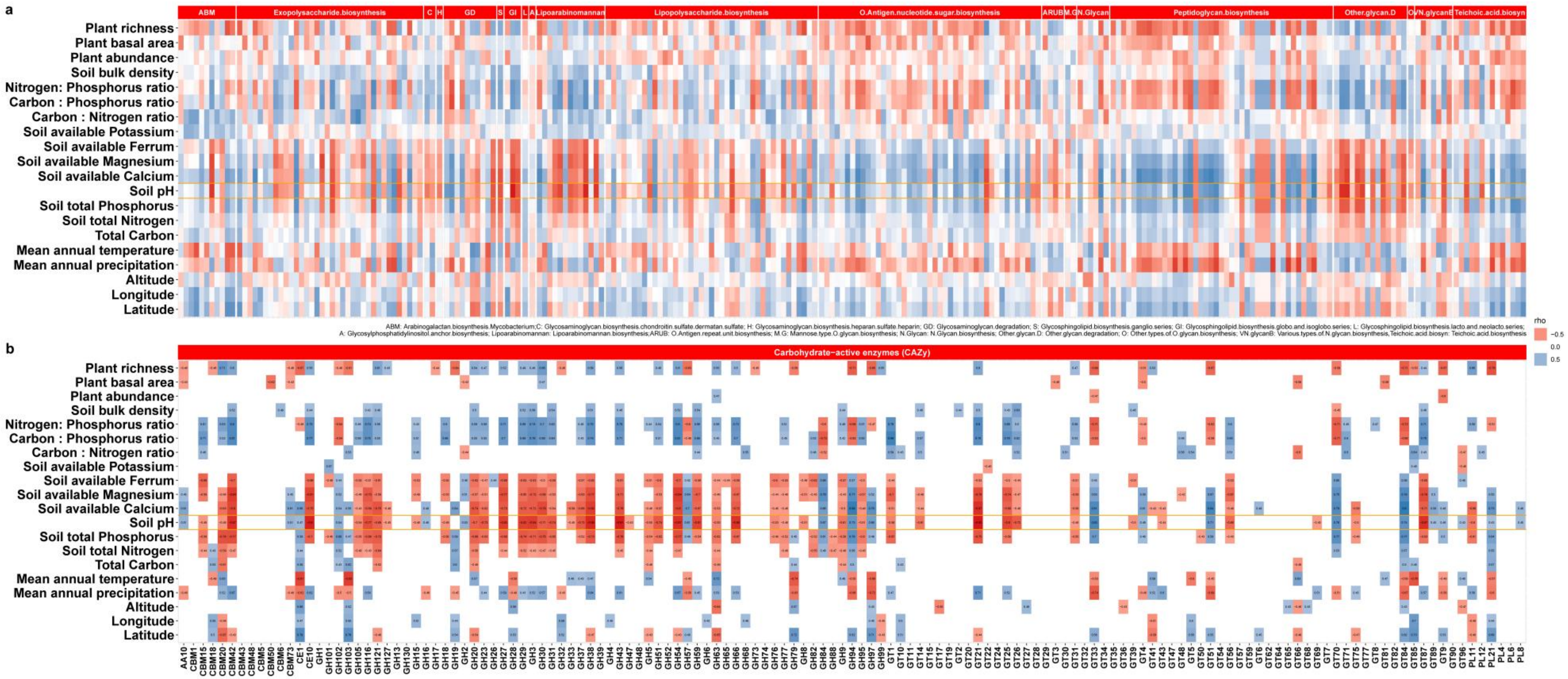

Supplementary Fig. 13. Genes of glycan biosynthesis and metabolism and carbohydrate-active enzymes (CAZy) associated with biotic and abiotic variables. a Heatmap showing positive (blue), and negative (red) Spearman's correlations (rho) between environmental variables (left) and relative abundance of genes of glycan biosynthesis and metabolism. **b** Heatmap showing positive (blue), and negative (red) Spearman's correlations (rho) between environmental variables (left) and relative abundance of genes of carbohydrate-active enzymes. Non-significant correlations are colored white. n = 36 samples. Source data are provided as a Source Data file.

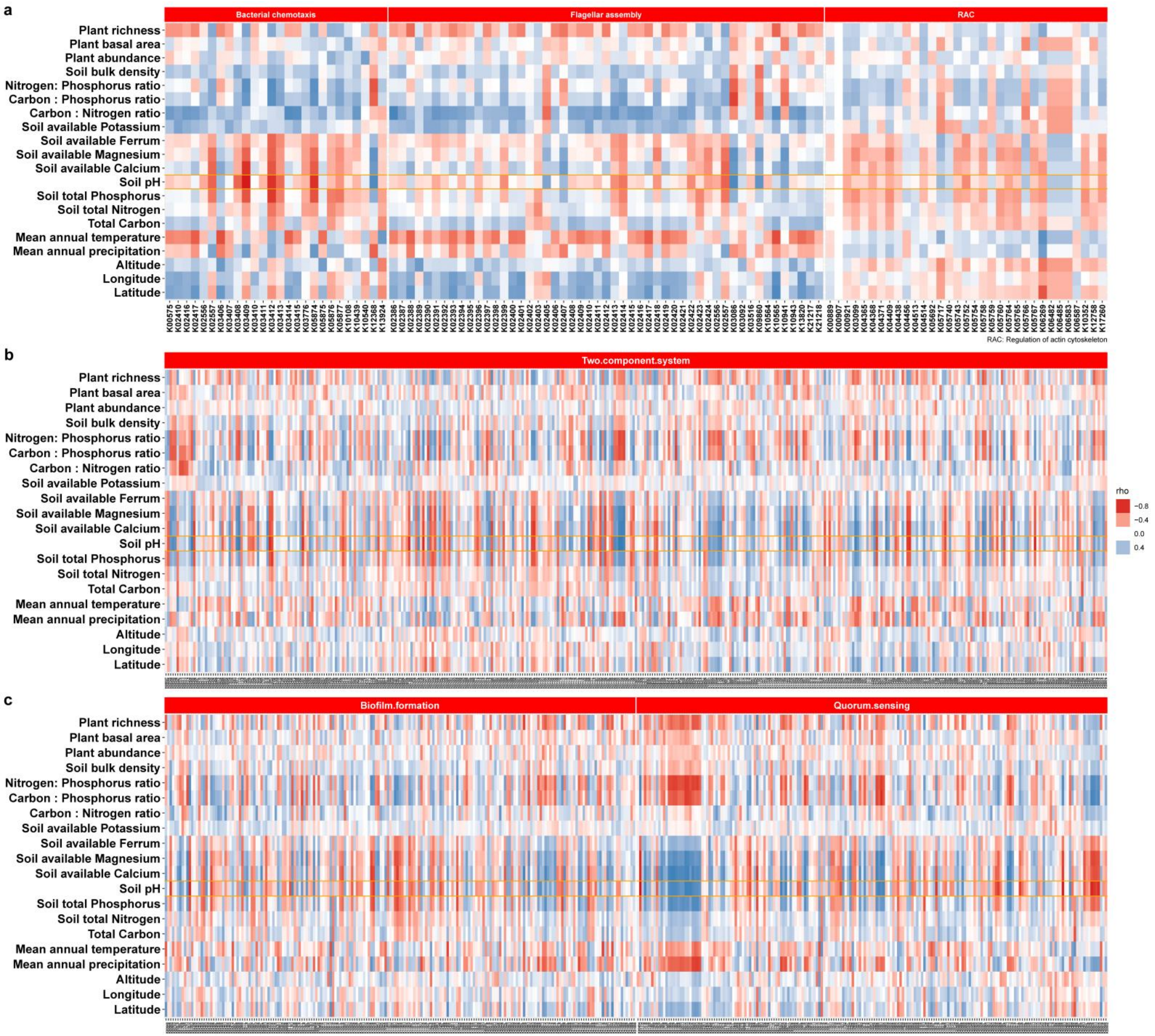
a
Bacterial chemotaxis
Flagellar assembly
RAC
Plant richness
Plant basal area
Plant abundance
Soil bulk density
Nitrogen: Phosphorus ratio
Carbon : Phosphorus ratio
Carbon : Nitrogen ratio
Soil available Potassium
Soil available Ferrum
Soil available Magnesium
Soil available Calcium
Soil pH
Soil total Phosphorus
Soil total Nitrogen
Total Carbon
Mean annual temperature
Mean annual precipitation
Altitude
Longitude
Latitude
RAC: Regulation of actin cytoskeleton
b
Two.component.system
Plant richness
Plant basal area
Plant abundance
Soil bulk density
Nitrogen: Phosphorus ratio
Carbon : Phosphorus ratio
Carbon : Nitrogen ratio
Soil available Potassium
Soil available Ferrum
Soil available Magnesium
Soil available Calcium
Soil pH
Soil total Phosphorus
Soil total Nitrogen
Total Carbon
Mean annual temperature
Mean annual precipitation
Altitude
Longitude
Latitude
rho
−0.8
−0.4
0.0
0.4
c
Biofilm.formation
Quorum.sensing
Plant richness
Plant basal area
Plant abundance
Soil bulk density
Nitrogen: Phosphorus ratio
Carbon : Phosphorus ratio
Carbon : Nitrogen ratio
Soil available Potassium
Soil available Ferrum
Soil available Magnesium
Soil available Calcium
Soil pH
Soil total Phosphorus
Soil total Nitrogen
Total Carbon
Mean annual temperature
Mean annual precipitation
Altitude
Longitude
Latitude

Supplementary Fig. 14. Genes of cell motility, signal transduction and cellular community associated with biotic and abiotic variables. **a** Heatmap showing positive (blue), and negative (red) Spearman's correlations (rho) between environmental variables (left) and relative abundance of genes of cell motility. **b** Heatmap showing positive (blue), and negative (red) Spearman's correlations (rho) between environmental variables (left) and relative abundance of genes of signal transduction (tow component system). **c** Heatmap showing positive (blue), and negative (red) Spearman's correlations (rho) between environmental variables (left) and relative abundance of genes of cellular community. n = 36 samples. Source data are provided as a Source Data file.

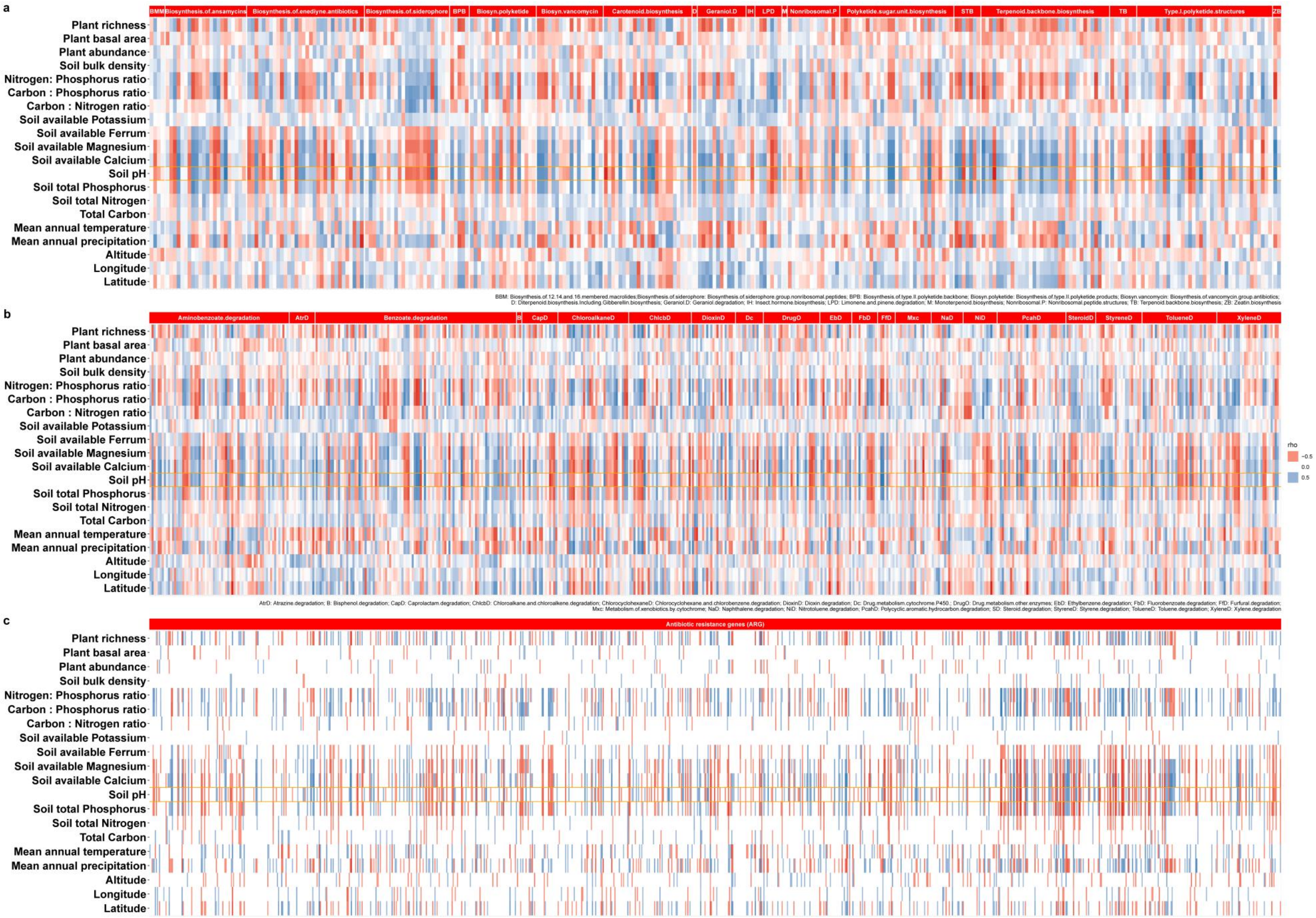

a
BBM
Biosynthesis.of.ansamycins
Biosynthesis.of.enediyne.antibiotics
Biosynthesis.of.siderophore
BPB
Biosyn.polyketide
Biosyn.vancomycin
Carotenoid.biosynthesis
D
Geraniol.D
IH
LPD
M
Nonribosomal.P
Polyketide.sugar.unit.biosynthesis
STB
Terpenoid.backbone.biosynthesis
TB
Type.I.polyketide.structures
ZB
Plant richness
Plant basal area
Plant abundance
Soil bulk density
Nitrogen: Phosphorus ratio
Carbon : Phosphorus ratio
Carbon : Nitrogen ratio
Soil available Potassium
Soil available Ferrum
Soil available Magnesium
Soil available Calcium
Soil pH
Soil total Phosphorus
Soil total Nitrogen
Total Carbon
Mean annual temperature
Mean annual precipitation
Altitude
Longitude
Latitude
BBM: Biosynthesis.of.12.14.and.16.membered.macrolides;Biosynthesis.of.siderophore: Biosynthesis.of.siderophore.group.nonribosomal.peptides; BPB: Biosynthesis.of.type.II.polyketide.backbone; Biosyn.polyketide: Biosynthesis.of.type.II.polyketide.products; Biosyn.vancomycin: Biosynthesis.of.vancomycin.group.antibiotics;
D: Diterpenoid.biosynthesis.Including.Gibberellin.biosynthesis; Geraniol.D: Geraniol.degradation; IH: Insect.hormone.biosynthesis; LPD: Limonene.and.pinene.degradation; M: Monoterpenoid.biosynthesis; Nonribosomal.P: Nonribosomal.peptide.structures; TB: Terpenoid.backbone.biosynthesis; ZB: Zeatin.biosynthesis
b
Aminobenzoate.degradation
AtrD
Benzoate.degradation
B
CapD
ChloroalkaneD
ChlcbD
DioxinD
Dc
DrugO
EbD
FbD
FfD
Mxc
NaD
NiD
PcahD
SteroidD
StyreneD
TolueneD
XyleneD
Plant richness
Plant basal area
Plant abundance
Soil bulk density
Nitrogen: Phosphorus ratio
Carbon : Phosphorus ratio
Carbon : Nitrogen ratio
Soil available Potassium
Soil available Ferrum
Soil available Magnesium
Soil available Calcium
Soil pH
Soil total Phosphorus
Soil total Nitrogen
Total Carbon
Mean annual temperature
Mean annual precipitation
Altitude
Longitude
Latitude
rho
−0.5
0.0
0.5
AtrD: Atrazine.degradation; B: Bisphenol.degradation; CapD: Caprolactam.degradation; ChlcbD: Chloroalkane.and.chloroalkene.degradation; ChlorocyclohexaneD: Chlorocyclohexane.and.chlorobenzene.degradation; DioxinD: Dioxin.degradation; Dc: Drug.metabolism.cytochrome.P450.; DrugO: Drug.metabolism.other.enzymes; EbD: Ethylbenzene.degradation; FbD: Fluorobenzoate.degradation; FfD: Furfural.degradation;
Mxc: Metabolism.of.xenobiotics.by.cytochrome; NaD: Naphthalene.degradation; NiD: Nitrotoluene.degradation; PcahD: Polycyclic.aromatic.hydrocarbon.degradation; SD: Steroid.degradation; StyreneD: Styrene.degradation; TolueneD: Toluene.degradation; XyleneD: Xylene.degradation
c
Antibiotic resistance genes (ARG)
Plant richness
Plant basal area
Plant abundance
Soil bulk density
Nitrogen: Phosphorus ratio
Carbon : Phosphorus ratio
Carbon : Nitrogen ratio
Soil available Potassium
Soil available Ferrum
Soil available Magnesium
Soil available Calcium
Soil pH
Soil total Phosphorus
Soil total Nitrogen
Total Carbon
Mean annual temperature
Mean annual precipitation
Altitude
Longitude
Latitude

Supplementary Fig. 15. Genes of metabolism of terpenoids and polyketides, xenobiotics biodegradation and metabolism and antibiotic resistance genes associated with biotic and abiotic variables. **a** Heatmap showing positive (blue), and negative (red) Spearman's correlations (rho) between environmental variables (left) and relative abundance of genes of metabolism of terpenoids and polyketides. **b** Heatmap showing positive (blue), and negative (red) Spearman's correlations (rho) between environmental variables (left) and relative abundance of genes of xenobiotics biodegradation and metabolism. **c** Heatmap showing positive (blue), and negative (red) Spearman's correlations (rho) between environmental variables (left) and relative abundance of genes of antibiotic resistance. Non-significant correlations are colored white. n = 36 samples. Source data are provided as a Source Data file.

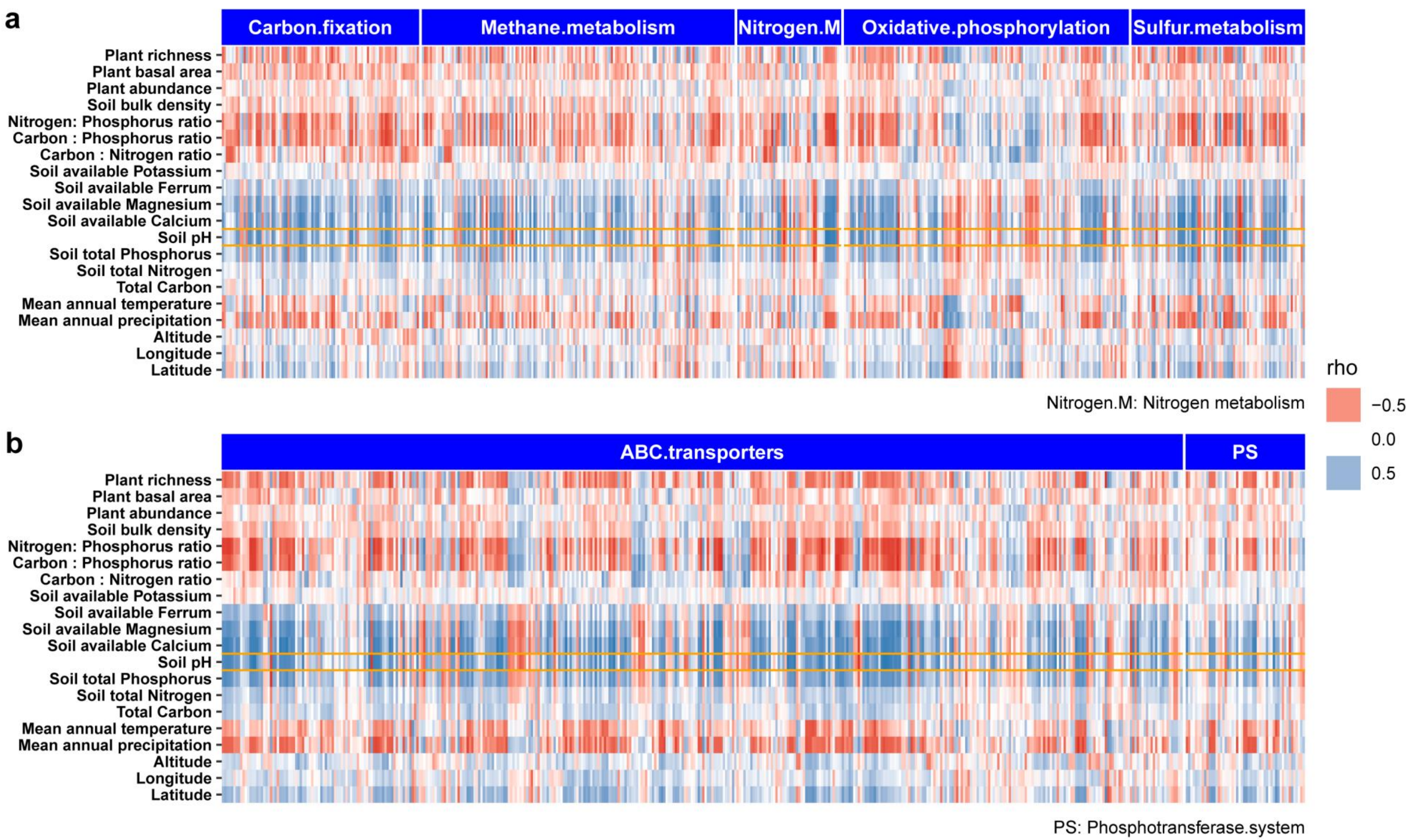

Supplementary Fig. 16. Prevailing of positive associations between pH and genes of energy metabolism and membrane transport. a Heatmap showing positive (blue), and negative (red) Spearman's correlations (rho) between environmental variables (left) and relative

abundance of genes of energy metabolism. **b** Heatmap showing positive (blue), and negative (red) Spearman's correlations (rho) between environmental variables (left) and relative abundance of genes of membrane transport. n = 36 samples. Source data are provided as a Source Data file.

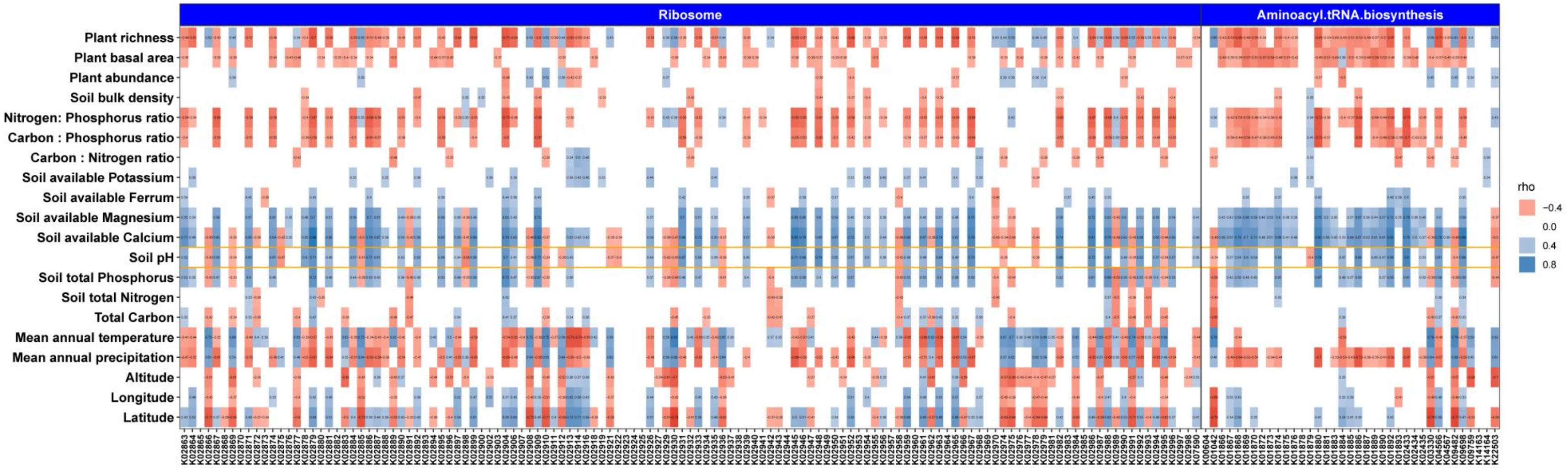

Supplementary Fig. 17. Heatmap showing positive (blue), and negative (red) Spearman's correlations (rho) between environmental variables (left) and relative abundance of genes involved in translation function. Non-significant correlations are colored white. n = 36 samples. Source data are provided as a Source Data file.

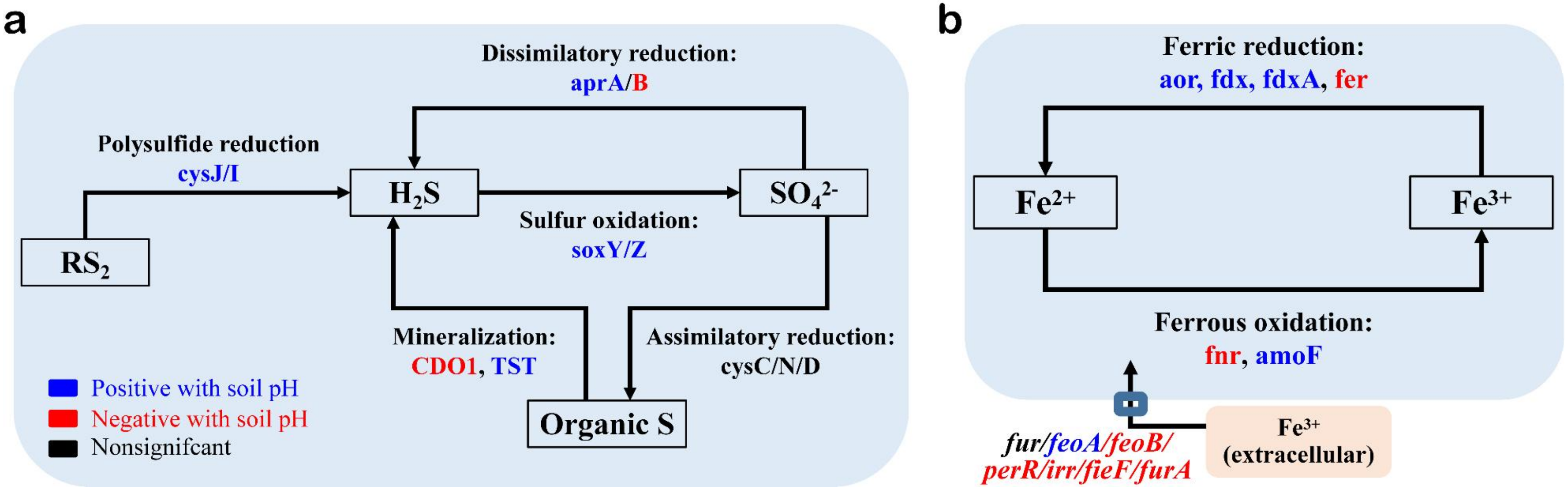

Supplementary Fig. 18. Genes involving biogeochemical cycle associated with environmental variables. a Six sulfur cycle genes positively correlate with soil pH, as compared to that two sulfur cycle genes negatively correlate with soil pH. **b** Five Fe cycle genes positively correlate with soil pH, and five iron transport genes negatively correlate with soil pH. Source data are provided as a Source Data file.

Supplementary Table 1 A list of publications on the relationship between pH and microbial diversity

Reference	Sampling	Scale	pH range	Curve	Method	Note
1	98 samples; Cross biome	North and South America	3.5 -8.5	Peak in neutral	16S-tRFLP	
2	88 sites (subset of 1)	North and South America	3.5-8.5	Peak in neutral	16S-454seq	
3	13 samples; Wetland	Carolina coastal plain	3.5 - 8.5	Peak in neutral	16S-clone-Seq	
4	7 samples	Qiantang River	6.0-8.0	Increase	16S-454seq	
5	20 samples	Tibetan forest	4.5-6.1	Increase	18S-454seq	Fungi
6	110 sites	China forests		?		No pH ~ diversity
7	17 sediments	15 Tibetan alkaline lake	7-10.5	Decrease	16S-454seq	
8	16 samples?	200m scale	4-8	Increase	16S-454seq	
9	36 samples	Soils from a pH gradient with lime addition in agricultural region of southern Brazil	4-6	Increase	16S-Illumina	Simpson
10	30 samples	Altitude gradient (10 sites)	3.5-7	Increase	18S-Illumina	Fungi
11	18 samples	Tibetan alkaline soils	7.3-9.3	Increase	16S-Illumina	
12			3.5-9.5		16S-Illumina	Stochasticity
13						Stochasticity
14						no pH ~ diversity
15	6 chronosequences (high productivity system)	Meta-analysis	3.5-9	Peak in neutral	16S-Illumina	
16	Dilution					Stochasticity
17	Global, 237 locations				16S-Illumina, Shotgun?	Network, pH cluster
18	179 sites, six ecosystem types,	Scotland	-	Increase	16S-454seq	Stoichiometry, TC, N:P, C:N,

	Scotland					C:P
19	Global	metanalysis	3.1-10.4			
20	France, 2173 soils		3.7-9.2			No pH ~ diversity
21	16 samples across biome	Desert, tundra, prairie, boreal, temperate and tropical forests	4.12-9.95	Increase	16S-Shotgun	
22	Guangdong, 18 samples	Three subtropical forests with two soil layers	3.6-4.5	Negative	16S- Illumina	H’ negative pH
23	Zhejiang, China, 95 samples	Bulk soil and rizhospheric soil in forests with different age	3.9-6.5	Increase	16S- Illumina	
24					16s	pH with different phyla
25	China	Meta-analysis, 47 forests and 105 samples	4.19-8.74	Increase	16S	
26	China, 343 soil samples	Five natural mountain forests across eastern China	3.8-8.5	Increase, peak at pH =7	16S-Illumina	Assembly process
27	Ningbo, China, 30 samples	pH gradient by adding CaCO3	4.62-7.46	Increase	16S-Illumina	
28	Tibet, China, 72 samples	Upper and deeper soil in wetland	5-9	Negative	16S-Illumina	
29	Australia, 16 samples	Four 78-years old plantations with different tree species	4.49-6.01	Increase	16S-Illumina	
30	29 samples	Agricultural soils	4.26-8.43	Increase	16S-Illumina	pH with different phyla
31	40 samples	Grassland soil with nitrogen addition	4.0-7.5	Increase	16S-454seq	
32	38 samples	Soils from oil refineries	7.5-9.5	Increase	16S- Illumina	
33	115 mineral soil samples	Typical forests across north	4.0-7.5	Increase	16S- Illumina	pH with different phyla

		and south China with the latitudes ranging from 18.70◦N to 51.53◦N				
34	15 samples	Temperate wheat land with fertilizations	4.64-6.36	Increase (ns)	16S- Illumina	
35	7 samples	Sediment of freshwater river	6.0-8.25	Increase	16S-454seq	
36	26 samples	Soils across the black soil zone of northeast China	4.56-6.57	Increase	16S-454seq	
37	14 samples	soil samples from a defined agricultural soil pH gradient in Craibstone, Scotland	4.5-7.5	Increase	16S- Illumina	
38		Water samples from river		Increase	16S- Illumina	Simpson
39	24 samples	Temperate forest soils with altitudinal gradient from 530 to 2200 m in Changbai Mountain	3.8-6.5	Increase	16S-454seq	pH with different phyla
40	1010 samples	Soil samples across the UK	3-9	Increase	16S-TRFLP- BigDye v3.1 chemistry	Simpson
41	Local		3.6-7	Increase	16S	
42	Local	12 soil samples with contrasting land cover				Negative correlation between pH and Acidobacteria
43	Global scale	615 composite topsoil samples from 151 locations from all continents and 23 countries	3.49-9.54 pH=2.99 for one sample	Increase	16S	

44	Global scale	30 studies and 1,998 samples from 21 countries				Negative correlation between pH and Acidobacteria
45	Global scale	3986 samples mostly collected from soil and fresh water	4-12<br>pH=2.0 for one sample	Peak near neutral pH (around 7)	16S	16S rRNA gene copy number
46	Global scale	Samples of top soils from global drylands, Americans, Australia, China, New South Wales	4.00-9.60	Increase	16S	
47		15 soil samples with contrasting land use	3.8-7.75	Increase	16S	
48	Global scale	237 sites globally distributed across arid, continental and temperate climates	4.00-9.00	Arid and Temperate: increase<br>Global: peak at around pH=7	16S	

Supplementary Table 2 Associations of certain microbial functions in this study*, in Ramoneda et al 2023 and in Malik et al 2018

This study (Wang et al)		Ramoneda et al 2023		Malik et al 2018	
**Function (KEGG Level3 or gene)**	**pH association**	**Function (pfam)**	**pH association**	**Function**	**pH association**
Carbon metabolism	(+)	Sugar metabolism	(+)	Carbon metabolism	(+)
Carbon fixation	(+)				
Glyoxylate and dicarboxylate metabolism	(+)				
Citrate cycle	(+)	Citrate transporter	(+)		
Methane metabolism	(+)				
ABC transporters	(+)	Transmembrane cation transporters/ Transmembrane anion transporter/Na+/H+ antiporters	(+)	ABC transporters	(+)
Metabolism of other amino acids	(+)	Methionine metabolism	(+)	Biosynthesis of amino acids	(+)
Oxidative phosphorylation	(+)			Oxidative phosphorylation	(+)
Ribosome	(+)			Ribosome	(+)
Flagellar assembly	(-)	Motility	(-)		
Metabolism of terpenoids polyketides	(-)				
Xenobiotics biodegradation metabolism	(-)	Phenol degradation	(-)		
Glycan biosynthesis metabolism	(-)				
Bacterial secretion system	(-)	Type IV secretion system	(-)		
Bacterial chemotaxis	(-)				
Two component system	(-)	Transmembrane proteins	(-)		
Porphyrin metabolism	(-)				
Biosynthesis of siderophore	(-)				
Lipopolysaccharide biosynthesis	(-)				

MTHFD; methylenetetrahydrofolate dehydrogenase	(+)	Folate metabolism	(+)		
ATPase family AAA domain-containing protein 3A/B	(+)	ATPases/AAA_25	(+)		
uvrA/B,excinuclease ABC subunit B	(+)	UV damage repair endonuclease	(+)		
ACH1; acetyl-CoA hydrolase [EC:3.1.2.1]	(-)	Acetyl-CoA hydrolase/transferase	(-)		
pufC; photosynthetic reaction center cytochrome c subunit	(-)	Cytochrome C photosynthetic reaction center	(-)		
dexA; dextranase	(-)	Hydrolases of dextrans Sugar metabolism	(-)		
hipA/B	(-)	Kinase that inhibits tRNA synthase (antibiosis)	(-)		
hypF; hydrogenase maturation protein HypF	(-)	Hydrogenase maturation	(-)		
csxA; exo-1,4-beta-D-glucosaminidase [EC:3.2.1.165]	(-)	Glucosaminidase	(-)		
NAGLU; alpha-N-acetylglucosaminidase [EC:3.2.1.50]	(-)				
kdpA/B/C	(-)	K^+ transporter	(-)		
rhtB; homoserine/homoserine lactone efflux protein	(-)	Acylated homoserine lactone metabolism	(-)		
scrY; sucrose porin	(-)	Carbohydrate porin	(-)		
TC.FEV.OM3, tbpA, hemR, lbpA, hpuB, bhuR, hugA, hmbR; hemoglobin/transferrin/lactoferrin receptor protein	(-)	Transferrin dimerization domain	(-)		
plc; phospholipase C [EC:3.1.4.3]	(-)	Phospholipase C	(-)		
K14645; serine protease [EC:3.4.21.-]	(+)	Serine protease	(-)		
ccdA; cytochrome c-type biogenesis protein	(-)	Motif part of C type cytochrome	(-)		
napC; cytochrome c-type protein NapC	(+)				

ccmF; cytochrome c-type biogenesis protein CcmF	(+)				
nrfE; cytochrome c-type biogenesis protein NrfE	(+)				
perR; Fur family transcriptional regulator, peroxide stress response regulator	(-)	Stress response	(-)		
furA; Fur family transcriptional regulator, stress-responsive regulator	(-)				
universal stress protein A/G/F/E	(+)				
lnuA_C_D_E, lin; lincosamide nucleotidyltransferase A/C/D/E	(+)	Nucleotidyltransferase	(-)		
pnp, PNPT1; polyribonucleotide nucleotidyltransferase [EC:2.7.7.8]	(+)				
				Purine metabolism	(+)
				RNA degradation	(-)
		Photolyase	(+)		
		Endonuclease/Exonuclease/phosphatase family	(+)		
		Lyase of methionine metabolism	(+)		
		Methyltransferases	(+)		
		Kinase that does AMPylation to proteins	(+)		
		Ca-dependent nuclease	(+)		
		Heme binding proteins	(+)		
		Gluc to Fruc 6-phosphate	(+)		
		Kinase that does AMPylation to proteins	(+)		
		Ca-dependent nuclease	(+)		
		Heme binding proteins	(+)		
		Ig like domains	(-)		
		Phosphatases in polysaccharide synthesis	(-)		

		Cytidylate kinase	(-)		
		Dehydrokinase	(-)		
		Helix-turn-helix domain	(-)		
		Lipid hydratase	(-)		
		Quinolones	(-)		
		Thyamin pirophsophate binding domain	(-)		
		Fe receptors	(-)		
		Lipid hydratase	(-)		

*Note a list of all KOs significantly associated with soil pH of our dataset as detected by threshold indicator analysis (TITAN) is provided in Supplementary Data 2.

Supplementary References

1 Fierer, N. & Jackson, R. B. The diversity and biogeography of soil bacterial communities. *Proc. Natl. Acad. Sci. USA.* **103**, 626-631 (2006). https://doi.org:10.1073/pnas.0507535103

2 Lauber, C. L., Hamady, M., Knight, R. & Fierer, N. Pyrosequencing-Based Assessment of Soil pH as a Predictor of Soil Bacterial Community Structure at the Continental Scale. *Appl. Environ. Microb.* **75**, 5111-5120 (2009). https://doi.org:doi:10.1128/AEM.00335-09

3 Hartman, W., Richardson, C., Vilgalys, R. & Bruland, G. Environmental and anthropogenic control of bacterial communities in wetland soils. *Proc. Natl. Acad. Sci. USA.* **105**, 17842-17847 (2008). https://doi.org:10.1073/pnas.0808254105

4 Hao, K. *et al.* pH levels drive bacterial community structure in the Qiantang River as determined by 454 pyrosequencing. *Front. Microbiol.* **6**, 285 (2015). https://doi.org:10.3389/fmicb.2015.00285

5 Wang, J.-T. *et al.* Soil pH determines the alpha diversity but not beta diversity of soil fungal community along altitude in a typical Tibetan forest ecosystem. *J. Soil. Sediment.* **15**, 1224-1232 (2015). https://doi.org:10.1007/s11368-015-1070-1

6 Ma, B. *et al.* Distinct biogeographic patterns for archaea, bacteria, and fungi along the vegetation gradient at the continental scale in eastern China. *mSystems* **2**, e00174-00116 (2017). https://doi.org:10.1128/mSystems.00174-16

7 Xiong, J. *et al.* Geographic distance and pH drive bacterial distribution in alkaline lake sediments across Tibetan Plateau. *Environ. Microbiol.* **14**, 2457-2466 (2012). https://doi.org:https://doi.org/10.1111/j.1462-2920.2012.02799.x

8 Rousk, J. *et al.* Soil bacterial and fungal communities across a pH gradient in an arable soil. *ISME J* **4**, 1340-1351 (2010).

9 Lammel, D. R. *et al.* Direct and indirect effects of a pH gradient bring insights into the mechanisms driving prokaryotic community structures. *Microbiome* **6**, 106 (2018). https://doi.org:10.1186/s40168-018-0482-8

10 Liu, D., Liu, G.-h., li, C., Wang, J. & Zhang, L. Soil pH determines fungal diversity along an elevation gradient in Southwestern China. *Sci. China Life Sci.* **61** (2018). https://doi.org:10.1007/s11427-017-9200-1

11 Shen, C. *et al.* Soil pH dominates elevational diversity pattern for bacteria in high elevation alkaline soils on the Tibetan Plateau. *FEMS Microbiol. Ecol.* **95** (2018). https://doi.org:10.1093/femsec/fiz003

12 Tripathi, B. M. *et al.* Soil pH mediates the balance between stochastic and deterministic assembly of bacteria. *ISME J.* **12**, 1072-1083 (2018). https://doi.org:10.1038/s41396-018-0082-4

13 Jiao, S. & Lu, Y. Soil pH and temperature regulate assembly processes of abundant and rare bacterial communities in agricultural ecosystems. *Environ. Microbiol.* **22**, 1052-1065 (2020). https://doi.org:https://doi.org/10.1111/1462-2920.14815

14 Malik, A. A. *et al.* Land use driven change in soil pH affects microbial carbon cycling processes. *Nat. Commun.* **9**, 3591 (2018). https://doi.org:10.1038/s41467-018-05980-1

15 Delgado-Baquerizo, M. *et al.* Changes in belowground biodiversity during ecosystem development. *Proc. Natl. Acad. Sci. USA.* **116**, 6891-6896 (2019). https://doi.org:doi:10.1073/pnas.1818400116

16 Xun, W. *et al.* Diversity-triggered deterministic bacterial assembly constrains community functions. *Nat. Commun.* **10**, 3833 (2019). https://doi.org:10.1038/s41467-019-11787-5
17 Delgado-Baquerizo, M. *et al.* A global atlas of the dominant bacteria found in soil. *Science* **359**, 320-325 (2018). https://doi.org:10.1126/science.aap9516
18 Delgado-Baquerizo, M. *et al.* It is elemental: soil nutrient stoichiometry drives bacterial diversity. *Environ. Microbiol.* **19**, 1176-1188 (2017). https://doi.org:https://doi.org/10.1111/1462-2920.13642
19 Delgado-Baquerizo, M. *et al.* Carbon content and climate variability drive global soil bacterial diversity patterns. *Ecol. Monogr.* **86**, 373-390 (2016). https://doi.org:https://doi.org/10.1002/ecm.1216
20 Karimi, B. *et al.* Biogeography of soil bacteria and archaea across France. *Sci. Adv.* **4**, eaat1808 (2018). https://doi.org:doi:10.1126/sciadv.aat1808
21 Fierer, N. *et al.* Cross-biome metagenomic analyses of soil microbial communities and their functional attributes. *Proc. Natl. Acad. Sci. USA.* **109**, 21390-21395 (2012). https://doi.org:10.1073/pnas.1215210110
22 Wang, Y.-F. *et al.* Key factors shaping prokaryotic communities in subtropical forest soils. *Appl. Soil Ecol.* **169** (2022). https://doi.org:10.1016/j.apsoil.2021.104162
23 Wang, B. *et al.* Factors driving the assembly of prokaryotic communities in bulk soil and rhizosphere of Torreya grandis along a 900-year age gradient. *Sci. Total Environ.* **837**, 155573 (2022). https://doi.org:10.1016/j.scitotenv.2022.155573
24 Muneer, M. A. *et al.* Soil pH: a key edaphic factor regulating distribution and functions of bacterial community along vertical soil profiles in red soil of pomelo orchard. *BMC Microbiol.* **22**, 38 (2022). https://doi.org:10.1186/s12866-022-02452-x
25 Yan, K., Dong, Y., Gong, Y., Zhu, Q. & Wang, Y. Climatic and edaphic factors affecting soil bacterial community biodiversity in different forests of China. *Catena* **207** (2021). https://doi.org:10.1016/j.catena.2021.105675
26 Ni, Y. *et al.* Soil pH determines bacterial distribution and assembly processes in natural mountain forests of eastern China. *Global Ecol. Biogeogr.* **30**, 2164-2177 (2021). https://doi.org:10.1111/geb.13373
27 Li, H. Q. *et al.* Soil pH has a stronger effect than arsenic content on shaping plastisphere bacterial communities in soil. *Environ. Pollut.* **287**, 117339 (2021). https://doi.org:10.1016/j.envpol.2021.117339
28 Kang, E. *et al.* Soil pH and nutrients shape the vertical distribution of microbial communities in an alpine wetland. *Sci. Total Environ.* **774** (2021). https://doi.org:10.1016/j.scitotenv.2021.145780
29 Zhou, X., Guo, Z., Chen, C. & Jia, Z. Soil microbial community structure and diversity are largely influenced by soil pH and nutrient quality in 78-year-old tree plantations. *Biogeosciences* **14**, 2101-2111 (2017). https://doi.org:10.5194/bg-14-2101-2017
30 Wu, Y., Zeng, J., Zhu, Q., Zhang, Z. & Lin, X. pH is the primary determinant of the bacterial community structure in agricultural soils impacted by polycyclic aromatic hydrocarbon pollution. *Sci. Rep.* **7**, 40093 (2017). https://doi.org:10.1038/srep40093

31 Zeng, J. *et al.* Nitrogen fertilization directly affects soil bacterial diversity and indirectly affects bacterial community composition. *Soil Biol. Biochem.* **92**, 41-49 (2016). https://doi.org:10.1016/j.soilbio.2015.09.018
32 Jiao, S. *et al.* Bacterial communities in oil contaminated soils: Biogeography and co-occurrence patterns. *Soil Biol. Biochem.* **98**, 64-73 (2016). https://doi.org:10.1016/j.soilbio.2016.04.005
33 Xia, Z. *et al.* Biogeographic distribution patterns of bacteria in typical Chinese forest soils. *Front. Microbiol.* **7**, 1106 (2016). https://doi.org:10.3389/fmicb.2016.01106
34 Zhou, J. *et al.* Influence of 34-years of fertilization on bacterial communities in an intensively cultivated black soil in northeast China. *Soil Biol. Biochem.* **90**, 42-51 (2015). https://doi.org:10.1016/j.soilbio.2015.07.005
35 Liu, S. *et al.* pH levels drive bacterial community structure in sediments of the Qiantang River as determined by 454 pyrosequencing. *Front. Microbiol.* **6**, 285 (2015). https://doi.org:10.3389/fmicb.2015.00285
36 Liu, J. *et al.* High throughput sequencing analysis of biogeographical distribution of bacterial communities in the black soils of northeast China. *Soil Biol. Biochem.* **70**, 113-122 (2014). https://doi.org:10.1016/j.soilbio.2013.12.014
37 Bartram, A. K. *et al.* Exploring links between pH and bacterial community composition in soils from the Craibstone Experimental Farm. *FEMS Microbiol. Ecol.* **87**, 403-415 (2014). https://doi.org:10.1111/1574-6941.12231
38 Staley, C. *et al.* Application of Illumina next-generation sequencing to characterize the bacterial community of the Upper Mississippi River. *J. Appl. Microbiol.* **115**, 1147-1158 (2013). https://doi.org:10.1111/jam.12323
39 Shen, C. *et al.* Soil pH drives the spatial distribution of bacterial communities along elevation on Changbai Mountain. *Soil Biol. Biochem.* **57**, 204-211 (2013). https://doi.org:10.1016/j.soilbio.2012.07.013
40 Griffiths, R. I. *et al.* The bacterial biogeography of British soils. *Environ. Microbiol.* **13**, 1642-1654 (2011). https://doi.org:10.1111/j.1462-2920.2011.02480.x
41 Zhalnina, K. *et al.* Soil pH Determines Microbial Diversity and Composition in the Park Grass Experiment. *Microb. Ecol.* **69**, 395-406 (2015). https://doi.org:10.1007/s00248-014-0530-2
42 Lauber, C. L., Strickland, M. S., Bradford, M. A. & Fierer, N. The influence of soil properties on the structure of bacterial and fungal communities across land-use types. *Soil Biol. Biochem.* **40**, 2407-2415 (2008). https://doi.org:10.1016/j.soilbio.2008.05.021
43 Guerra, C. A. *et al.* Global hotspots for soil nature conservation. *Nature* **610**, 693-698 (2022). https://doi.org:10.1038/s41586-022-05292-x
44 Ramirez, K. S. *et al.* Detecting macroecological patterns in bacterial communities across independent studies of global soils. *Nat. Microbiol.* **3**, 189-196 (2018). https://doi.org:10.1038/s41564-017-0062-x
45 Thompson, L. R. *et al.* A communal catalogue reveals Earth's multiscale microbial diversity. *Nature* **551**, 457-463 (2017). https://doi.org:10.1038/nature24621

46 Delgado-Baquerizo, M. *et al.* Palaeoclimate explains a unique proportion of the global variation in soil bacterial communities. *Nat. Ecol. Evol.* **1**, 1339-1347 (2017). https://doi.org:10.1038/s41559-017-0259-7

47 Terrat, S. *et al.* Meta-barcoded evaluation of the ISO standard 11063 DNA extraction procedure to characterize soil bacterial and fungal community diversity and composition. *Microb. Biotechnol.* **8**, 131-142 (2015). https://doi.org:10.1111/1751-7915.12162

48 Delgado-Baquerizo, M. & Eldridge, D. J. Cross-Biome Drivers of Soil Bacterial Alpha Diversity on a Worldwide Scale. *Ecosystems* **22**, 1220-1231 (2019). https://doi.org:10.1007/s10021-018-0333-2

49 Brewer, T.E., Handley, K.M., Carini, P., et al. (2017). Genome reduction in an abundant and ubiquitous soil bacterium '*Candidatus Udaeobacter copiosus*'. *Nat. Microbiol.* 2, 16198, 10.1038/nmicrobiol.2016.198.
