# Supplementary material for: Bacterial genome size and gene functional diversity negatively correlate with taxonomic diversity along a pH gradient
Source: Nat Commun. 2023 Nov 17;14:7437. doi: 10.1038/s41467-023-43297-w (PMC10656551; doi:10.1038/s41467-023-43297-w)
Supplement: Supplementary file 3 — Description of Additional Supplementary Information [file 41467_2023_43297_MOESM3_ESM.pdf]

Description of Additional Supplementary Files:

Supplementary Data 1: The numerical data for plotting Supplementary Fig. 1

Supplementary Data 2: Association between KOs and soil pH identified by threshold indicator taxa analysis (TITAN)
