# Supplementary material for: Bacterial genome size and gene functional diversity negatively correlate with taxonomic diversity along a pH gradient
Source: Nat Commun. 2023 Nov 17;14:7437. doi: 10.1038/s41467-023-43297-w (PMC10656551; doi:10.1038/s41467-023-43297-w)
Supplement: Supplementary file 6 — Reporting Summary [file 41467_2023_43297_MOESM6_ESM.pdf]

nature portfolio

Corresponding author(s): Cheng Gao

Last updated by author(s): Oct 13, 2023

Reporting Summary

Nature Portfolio wishes to improve the reproducibility of the work that we publish. This form provides structure for consistency and transparency in reporting. For further information on Nature Portfolio policies, see our Editorial Policies and the Editorial Policy Checklist.

Statistics

For all statistical analyses, confirm that the following items are present in the figure legend, table legend, main text, or Methods section.

n/a	Confirmed	
☐	☒	The exact sample size (*n*) for each experimental group/condition, given as a discrete number and unit of measurement
☐	☒	A statement on whether measurements were taken from distinct samples or whether the same sample was measured repeatedly
☐	☒	The statistical test(s) used AND whether they are one- or two-sided<br>*Only common tests should be described solely by name; describe more complex techniques in the Methods section.*
☒	☐	A description of all covariates tested
☐	☒	A description of any assumptions or corrections, such as tests of normality and adjustment for multiple comparisons
☐	☒	A full description of the statistical parameters including central tendency (e.g. means) or other basic estimates (e.g. regression coefficient) AND variation (e.g. standard deviation) or associated estimates of uncertainty (e.g. confidence intervals)
☐	☒	For null hypothesis testing, the test statistic (e.g. *F*, *t*, *r*) with confidence intervals, effect sizes, degrees of freedom and *P* value noted<br>*Give P values as exact values whenever suitable.*
☒	☐	For Bayesian analysis, information on the choice of priors and Markov chain Monte Carlo settings
☒	☐	For hierarchical and complex designs, identification of the appropriate level for tests and full reporting of outcomes
☐	☒	Estimates of effect sizes (e.g. Cohen's *d*, Pearson's *r*), indicating how they were calculated

Our web collection on statistics for biologists contains articles on many of the points above.

Software and code

Policy information about availability of computer code

Data collection	This study did not use any commercial, open source and custom code to collect data.
Data analysis	Meatbarcoding data processing: FastQC v0.11.5; USEARCH v8.0; cutadapt v1.9.1; Metagenome data processing: MEGAHIT v1.2.9; EukRep v0.6.7; VIBRANT v1.2.1; BBMap v39.01; Quast v5.2.0; gRodon v2.3.0; makeblastdb v2.13.0; BLAST v2.13.0; redundans; prodigal v2.6.3; CD-HIT v4.8.1; salmon v1.6.0; EggNOG-mapper tool; DIAMOND tool; Kaiju; Statistical analyses: R statistical programming language version 4.2.1; vegan v2.5.7; psych v2.2.9; igraph v1.3.5; linkET v0.0.7.1; TITAN2 v2.4.2; edgeR 3.38.4; nlme 3.1.160. The source code implementing the analyses in this manuscript is available on Github (https://github.com/FunCongWang/CForBio.metagenome).

For manuscripts utilizing custom algorithms or software that are central to the research but not yet described in published literature, software must be made available to editors and reviewers. We strongly encourage code deposition in a community repository (e.g. GitHub). See the Nature Portfolio guidelines for submitting code & software for further information.

Data

Policy information about availability of data

All manuscripts must include a data availability statement. This statement should provide the following information, where applicable:

- Accession codes, unique identifiers, or web links for publicly available datasets
- A description of any restrictions on data availability
- For clinical datasets or third party data, please ensure that the statement adheres to our policy

All metabarcoding and metagenomics sequences in this study have been deposited in the National Center for Biotechnology Information database (PRJNA986291

[https://www.ncbi.nlm.nih.gov/bioproject/PRJNA986291/]). The WorldClim database is available through [https://www.worldclim.org]. Source data are provided with this paper.

Research involving human participants, their data, or biological material

Policy information about studies with human participants or human data. See also policy information about sex, gender (identity/presentation), and sexual orientation and race, ethnicity and racism.

Reporting on sex and gender	not applicable
Reporting on race, ethnicity, or other socially relevant groupings	not applicable
Population characteristics	not applicable
Recruitment	not applicable
Ethics oversight	not applicable

Note that full information on the approval of the study protocol must also be provided in the manuscript.

Field-specific reporting

Please select the one below that is the best fit for your research. If you are not sure, read the appropriate sections before making your selection.

☐ Life sciences ☐ Behavioural & social sciences ☒ Ecological, evolutionary & environmental sciences

For a reference copy of the document with all sections, see nature.com/documents/nr-reporting-summary-flat.pdf

Ecological, evolutionary & environmental sciences study design

All studies must disclose on these points even when the disclosure is negative.

Study description	This study sampled soils from 12 forest sites covering temperate, subtropical and tropical climatic zones. In the forest of Genhe, soil samples were collected in long-term observation plots. In other 11 forests, the Chinese Forest Biodiversity Monitoring Network (CForBio) had established a permanent plot (15 to 25 ha) consisting of hundreds of 20 m × 20 m quadrats (375 to 625 quadrats). Three quadrats in each permanent plot were randomly selected for soil sampling in this study.
Research sample	This study includes 36 top soil samples (0-10 cm) from 12 forests in China: the Inner Mongolia Daxing'anling Forest Ecosystem Research Station (3 samples), Liangshui Experimental Forest Farm, the Research Station of Changbai Mountain Forest Ecosystem (3 samples), the Beijing Forest Experimental Station (3 samples), the Baotianman National Nature Reserve (3 samples), the East China Normal University Tiantong National Forest Ecosystem Observation and Research Station (3 samples), the Badagong Mountain National Nature Reserve (3 samples), the Zhejiang Qianjiangyuan Forest Biodiversity National Observation and Research Station (3 samples), the Heishiding Nature Reserve (3 samples), the Dinghu Mountain National Nature Reserve (3 samples), the Nonggang National Nature Reserve (3 samples), and the National Forest Ecosystem Research Station at Xishuangbanna (3 samples).<br>The 12 forest sites cover temperate, subtropical and tropical climatic zones with a wide range of soil pH (3.68-7.22). We apply soil sampling at these sites by oral application, and we have acknowledged relevant staff in the "Acknowledgement" section.<br>The metabarcoding and metagenome sequence data of these 36 soil samples in this study have been deposited in the National Center for Biotechnology Information database (PRJNA986291[https://www.ncbi.nlm.nih.gov/bioproject/PRJNA986291/]).
Sampling strategy	We selected 12 forests of China in this study, because 4 of them are representative temperate forest, 4 of them are representative subtropical forest, and 4 of them are representative tropical forest. Four replicates for each type of forest meet the basic requirement for statistical analyses. In each forest, three quadrats in each permanent plot were randomly selected for soil sampling in this study. Three randomly replicates for each forests meet the requirement for statistical analyses. In each quadrat, ten soil cores (10 cm in depth, 5 cm in diameter) were evenly collected and mixed to make one composite sample, resulting in a total of 36 soil samples (3 quadrats × 12 plots). Combing ten soil cores from one quadrats into one composite sample makes this sample can represent the quadrats.
Data collection	Latitude, longitude, soil bulk density, soil available cations and plant data (abundance, richness and basal area) of the study sites were provided by the CForBio organization or manager of the plot (Ke-Ping Ma, Min Cao, Liang Chen, Xiao-Yong Chen, Xiao-Bao Deng, Xiao-Jun Du, Zhan-Qing Hao, Ming-Xi Jiang, Guang-Ze Jin, Bu-Hang Li, Xian-Kun Li, Wei-Guo Sang, Wan-Hui Ye, Ming-Jian Yu, and Xiao-Liang Zhang). Latitude and longitude were recorded by GPS device. Soil bulk density was calculated as the dry weight of soil divided by its volume. Plant community data were surveyed by manual. The mean annual temperature (MAT) and mean annual precipitation (MAP) were obtained from the WorldClim database (www.worldclim.org) with a resolution of 2.5 min.
Timing and spatial scale	In each plot, we only sampled once during March to October, 2014. The 36 plots from 12 forests along a latitudinal gradient (N21.6°-N50.9°) in China.
Data exclusions	No data were excluded from the analyses.

Reproducibility	Our experiment used three replicate samples of each forest, and we confirm that all attempts at replications were successful.
Randomization	There are 12 sampling sites in this study. In each site, there are three replicates. The three replicates were selected randomly.
Blinding	Data collection and analysis were not blindly, because samples in this study were collected from field forests, and non-blind design would not affect the experimental results.

Did the study involve field work? ☒ Yes ☐ No

Field work, collection and transport

Field conditions	This study collected 36 soil samples from 12 forests, with mean annual temperature ranging from -4.7 °C to 22 °C, and with mean annual precipitation ranging from 500 mm to 2105.4mm.
Location	These 12 forests with latitude from N 21.61° to N 50.93°, longitude from E 101.58° to E 128.81°, and altitude from 245 m to 1682 m.
Access & import/export	We accessed these forests by connecting the manager of the forest experimental station. All sampling conducts were permitted by the Chinese Forest Biodiversity Monitoring Network Organization or the founder of the plot. We have acknowledged people and organizations in the manuscript. People: Ke-Ping Ma, Min Cao, Liang Chen, Xiao-Yong Chen, Xiao-Bao Deng, Xiao-Jun Du, Zhan-Qing Hao, Ming-Xi Jiang, Guang-Ze Jin, Bu-Hang Li, Xian-Kun Li, Wei-Guo Sang, Wan-Hui Ye, Ming-Jian Yu, and Xiao-Liang Zhang. Organizations: the Inner Mongolia Daxing'anling Forest Ecosystem Research Station, Liangshui Experimental Forest Farm, the Research Station of Changbai Mountain Forest Ecosystem, the Beijing Forest Experimental Station, the Baotianman National Nature Reserve, the East China Normal University Tiantong National Forest Ecosystem Observation and Research Station, the Badagong Mountain National Nature Reserve, the Zhejiang Qianjiangyuan Forest Biodiversity National Observation and Research Station, the Heishiding Nature Reserve, the Dinghu Mountain National Nature Reserve, the Nonggang National Nature Reserve, and the National Forest Ecosystem Research Station at Xishuangbanna.<br>We imported soils samples into our laboratory from field forest by using cool box. It is safe and does not affect society activity.
Disturbance	We avoided destroying plants and large animals during our soil sampling.

Reporting for specific materials, systems and methods

We require information from authors about some types of materials, experimental systems and methods used in many studies. Here, indicate whether each material, system or method listed is relevant to your study. If you are not sure if a list item applies to your research, read the appropriate section before selecting a response.

Materials & experimental systems

n/a	Involved in the study
☒	☐ Antibodies
☒	☐ Eukaryotic cell lines
☒	☐ Palaeontology and archaeology
☒	☐ Animals and other organisms
☒	☐ Clinical data
☒	☐ Dual use research of concern
☒	☐ Plants

Methods

n/a	Involved in the study
☒	☐ ChIP-seq
☒	☐ Flow cytometry
☒	☐ MRI-based neuroimaging
